# Synthesis and Photophysical Properties of T‐Shaped Coinage‐Metal Complexes

**DOI:** 10.1002/chem.202000726

**Published:** 2020-05-15

**Authors:** George Kleinhans, Alan K.‐W. Chan, Ming‐Yi Leung, David C. Liles, Manuel A. Fernandes, Vivian W.‐W. Yam, Israel Fernández, Daniela I. Bezuidenhout

**Affiliations:** ^1^ Chemistry Department University of Pretoria Private X20 Hatfield 0028 South Africa; ^2^ Molecular Sciences Institute School of Chemistry University of the Witwatersrand Johannesburg 2050 South Africa; ^3^ Institute of Molecular Functional Materials and Department of Chemistry The University of Hong Kong Pokfulam Road Hong Kong P. R. China; ^4^ Departamento de Química Orgánica I Centro de Innovación en Química Avanzado (ORFEO-CINQA) and Facultad de Ciencias Químicas Universidad Complutense de Madrid 28040 Madrid Spain; ^5^ Laboratory of Inorganic Chemistry, Environmental and Chemical Engineering University of Oulu P. O. Box 3000 90014 Oulu Finland

**Keywords:** 1,2,3-triazol-5-ylidenes, absorption and emission properties, coinage metals, pincer complexes, T-shaped geometries

## Abstract

The photophysical properties of a series of T‐shaped coinage *d* ^10^ metal complexes, supported by a bis(mesoionic carbene)carbazolide (CNC) pincer ligand, are explored. The series includes a rare new example of a tridentate T‐shaped Ag^I^ complex. Post‐complexation modification of the Au^I^ complex provides access to a linear cationic Au^I^ complex following ligand alkylation, or the first example of a cationic square planar Au^III^−F complex from electrophilic attack on the metal centre. Emissions ranging from blue (Cu^I^) to orange (Ag^I^) are obtained, with variable contributions of thermally‐dependent fluorescence and phosphorescence to the observed photoluminescence. Green emissions are observed for all three gold complexes (neutral T‐shaped Au^I^, cationic linear Au^I^ and square planar cationic Au^III^). The higher quantum yield and longer decay lifetime of the linear gold(I) complex are indicative of increased phosphorescence contribution.

## Graphical abstract

**T‐Shaped coinage‐metal complexes**: Photoluminescence tuning mediated by a bis(mesoionic carbene)amido pincer ligand for *d* ^10^ complexes of unusual T‐shape, linear Au^I^ and square planar Au^III^.

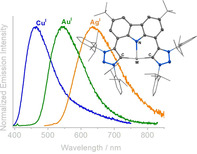

In the development of efficient organic light emitting devices (OLEDs), basic requirements of phosphorescent emitters include high external quantum efficiencies (EQE) of the emission, coupled with appropriate radiative lifetimes in the order of microseconds to facilitate the intersystem crossing (ISC) from the triplet to the singlet state.^1^ These specifications have been amply met using iridium(III) and platinum(II) emitters,^2^ with an increasing number of reports detailing the utility of the lesser explored gold(III) complexes. Indeed, CNC‐3 and CCN‐4 cyclometallated gold(III) complexes excelling in emission color tuning, solubility and thermal stability have recently yielded OLEDs with very high EQEs and similarly long device operational half‐lifetimes.4b Lower oxidation state gold(I), and the other *d* ^10^ coinage metals, copper(I) and silver(I), have also been the focus of concurrent investigations into their use in OLEDs. One of the more successful design strategies on this front employs the linear bonding geometry of carbene‐metal‐amides (CMAs), in which all three *d* ^10^ metals (Cu^I^, Ag^I^ and Au^I^), have similarly accomplished excellent EQE performance and/or high brightness OLED operation, notably employing carbazolide derivatives as the donor amide partners with the acceptor carbenes.^5^ These CMAs can display particularly short (ns) emission lifetimes in thermally assisted delayed fluorescence (TADF), based on the rapid triplet‐to‐singlet ISC, unlike the heavy atom (metal) phosphorescent emitters relying on spin‐orbit coupling.1b, 6

The dependency of photoluminescence on the coordination geometry of *d* ^10^ coinage metal complexes is well‐known,^7^ for example, 3‐coordinate trigonal planar copper(I) complexes showed tunable behavior from pure phosphorescence to TADF depending on the carbene‐metal‐amine dihedral angles.7c Extending 3‐coordinate systems beyond trigonal planar geometries to a ground state Jahn Teller‐distorted T‐shape has been an early theoretical target for photophysical tuning of the singlet‐triplet gap.^8^ However, the availability of ligand scaffolds allowing such geometry is limited, and reports of T‐shaped group 11 metal(I) complexes are still very rare.^9^, ^10^, ^11^ We have previously reported a monoanionic CNC‐pincer ligand ([H_3_CNC]PF_6_⋅Cl, Figure 1), comprising a central carbazolide flanked by two diarylated 1,2,3‐triazol‐5‐ylidenes (trz),^12^, ^13^ as a strongly donor ligand capable of stabilizing such a three coordinate geometry with a ‘vacant’ coordination site, for both Cu^I^ (**1**)9c and Au^I^ (**3**)11f (Figure 1). Moreover, the unique remote basicity of the Au^I^ (occupied *d*‐orbital) enabled by both the strained T‐shape geometry and the strongly electron donating nature of the ligand, could be exploited for reactions with electrophiles at either the nucleophilic carbazolide amido (**4**, Figure 1) or the gold(I) center to yield the cationic [(CN^Me^C)Au^I^]^+^ or oxidized [(CNC)Au^III^R]^+^ (R=H, Me, CH_2_Cl) complexes, respectively.11f Thus, having a C^N^C pincer ligand scaffold that allows for both T‐shape *d* ^10^ coinage metals and square planar cyclometallated gold(III) complexes, we decided to expand the series of complexes to include also Ag^I^ (**2**, Figure 1) and Au^III^−F (**5**, Figure 1) for a preliminary investigation of their photophysical properties. These complexes combine the established luminescent motif of donor amides (especially carbazolide), mesoionic carbenes and 3‐coordinate T‐shape/cyclometallated square planar complexes of the coinage metals. The strong σ donor and poor π acceptor properties of the 1,2,3‐triazol‐5‐ylidenes, as an example of the class of mesoionic carbenes, are increasingly being utilized in studies exploring their photoluminescent applications.^14^

**Figure 1:**
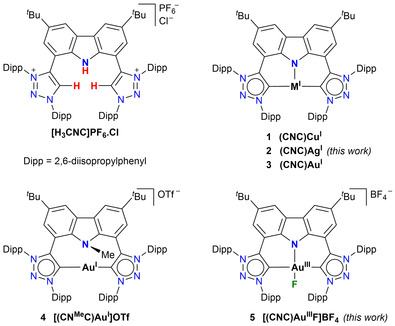
Precursor bis(triazolium)carbazole ligand salt [H_3_CNC]PF_6_⋅Cl and neutral (**1**–**3**) and cationic (**4**, **5**) metal complexes employed in this study.

Complex **2** was prepared directly from the reaction of the ligand precursor [H_3_CNC]PF_6_⋅Cl with excess Ag_2_O in the presence of KBr in CH_2_Cl_2_, in the absence of light (Scheme 1, see also Supporting Information, Section S2). The dark orange **2** is both thermally and atmospherically stable, and was characterized by NMR, HRMS spectroscopic and single crystal X‐ray diffraction techniques. Disappearance of the acidic carbazole and triazolium protons of [H_3_CNC]PF_6_⋅Cl in the ^1^H NMR spectrum of **2** (Figure S2), as well as the appearance of the carbene carbon atom doublet of doublets (*δ*_C_ 177.4, *J=*185.3 Hz, 13.2 Hz) in the ^13^C NMR spectrum (Figure S3), confirm the formation of **2**. A T‐shape geometry is unambiguously shown in the molecular structure obtained for a single crystal of **2** (Figure 2); only the third known example of a T‐shaped silver complex.^10^ Both the chemical resonance observed for the carbene carbon atom, as well as the carbene carbon‐silver bond lengths fall within range with that reported for known silver(I) triazolylidene complexes,14b, 15 whereas the rigid carbazole backbone deviates from planarity as seen also for rhodium(I) complexes of carbazolide‐pincer ligands.^16^

**Scheme 1:**
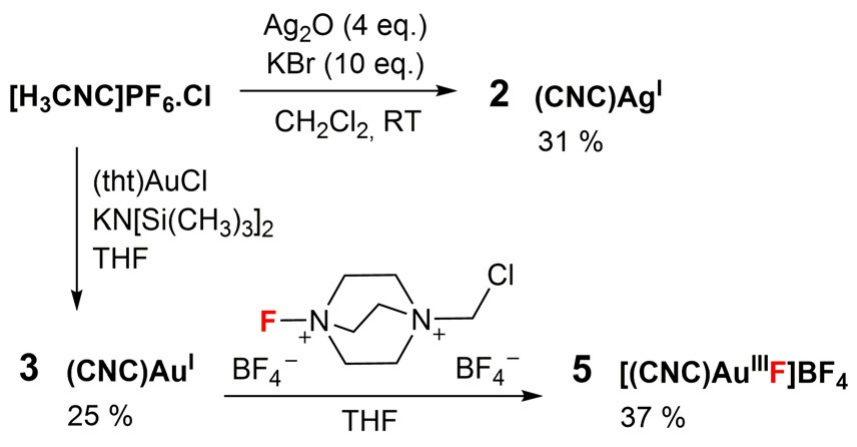
Preparation of new complexes **2** and **5**.

**Figure 2:**
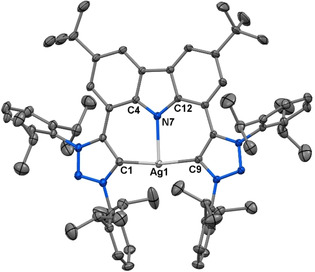
Solid‐state structure of **2**. Atomic displacement ellipsoids shown at the 50 % probability level. Hydrogen atoms omitted for clarity. Selected bond lengths [Å], bond angles [°] and torsion angles [°] for **2**: Ag−C_carbene_ 2.0988(12) and 2.1002(13), respectively; Ag−N_carbazol_ 2.2870(11); N_carbazol_–Ag–C_carbene_ 85.57(4) and 85.90(4), respectively; C_carbene_–Ag–C_carbene_ 171.35(5); C_carbazol_–N_carbazol_–Ag–C_carbene_ −177.6(1) and −174.8(1), respectively.^24^

The facile chemical oxidation of complex **3** by reaction with electrophilic protonating or alkylating agents,11f prompted us to determine the oxidation potential of this complex with cyclic voltammetry (CV). The CV experiments were carried out in solvent THF and [^*n*^Bu_4_N][PF_6_] as supporting electrolyte under inert atmospheric conditions, and referenced to the [Cp_2_Fe]^0/+1^ couple (Figure S1). A single two‐electron oxidation event was observed. Remarkably, the potential for the Au^I/III^ couple was determined to be *E* ^0^=−0.39 V, and at least quasi‐reversible with *E_pa_*−*E*_pc_=97 mV to confirm viability of mild chemical oxidation, in line with quantum theoretical studies.^17^ Thus, **5** was prepared by in situ deprotonation of [H_3_CNC]PF_6_⋅Cl and metalation to yield, after purification and isolation, complex **3** (Scheme 1). Subsequent treatment of **3** with a stoichiometric amount of the fluorinating agent Selectfluor in THF yielded **5** (37 % yield) (see Supporting Information, Section S2).

Well‐defined, isolable gold(III) fluorido complexes are not common, although they have been implicated as transient catalytic intermediates.^18^ In these reports, preparative conditions have not included the use of mercuric reagents and initial luminescence properties were investigated. Complex **5** was isolated as a lime‐green solid and is the first example of a stable, monomeric cationic gold(III) fluoride. The disappearance of the gold(I) carbene carbon resonance of **3** (*δ*_C_ 176.0 ppm) is consistent with an Au^I/III^ oxidation event, although the expected upfield shifted Au^III^ carbene carbon atom resonance was not detected. The ^19^F NMR spectrum displays fluorine resonances at *δ*_F_ −299.9 and −153.2 ppm, respectively, for the Au−F^−^ and BF_4_^−^ counterion. The fluoride resonance of −299.9 ppm is significantly upfield from neutral gold(III) fluorides (*δ*_F_ −255 ppm),18a most likely due to the cationic gold centre.

A single crystal X‐ray diffraction study unambiguously confirmed the formation of **5** (Figure 3). The short Au−N bond length of 2.000(6) Å resembles the short bond lengths of other gold(III) C^N^C complexes,11f with a gold(III)‐carbene carbon bond length of 2.033(5) Å. The Au−F bond length (1.947(5) Å) is comparable to the known bond lengths of the neutral gold(III) fluorides.^18^

**Figure 3:**
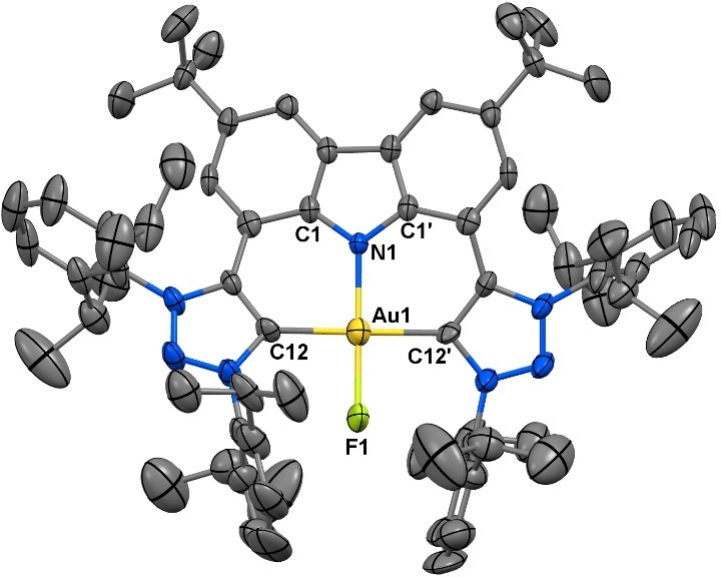
Solid‐state structure of **5**. Atomic displacement ellipsoids shown at the 50 % probability level. Hydrogen atoms and counter ion BF_4_^−^ are omitted for clarity. Selected bond lengths [Å], bond angles [°] and torsion angles [°] for **5**: Au−C_carbene_ 2.033(5); Au−N_carbazol_ 2.000(6); N_carbazol_–Au–C_carbene_ 89.61(14); C_carbene_–Au–C_carbene_ 179.2(3); C_carbazol_–N_carbazol_–Au–C_carbene_ 167.7(1).^24^

Again, distortion of the ligand scaffold (Figure 3) is observed to accommodate both the increased steric hindrance in the pincer pocket by the fluoride ligand, as well as the near‐perfect square planar geometry around the metal centre.

Photophysical data^19^ for the precursor [H_3_CNC]PF_6_⋅Cl and complexes **1**–**5** are summarized in Table 1, and their UV–Vis and emission spectra in various media are presented in Figures 4, 5 and 6 and the Supporting Information, Figures S6–S26. Both THF and acetonitrile have been used to explore the nature of the excited states at room temperature, and the corresponding analogous 2‐MeTHF and butyronitrile are used for the emission measurements at 77 K. Only complexes **2**–**4** were investigated in acetonitrile due to their insufficient solubility to reach concentrations appropriate for measurements at room temperature. The UV–Vis absorption spectra for the complexes in THF are shown in Figure 4. The lowest energy absorption bands correspond to the intraligand charge transfer (ILCT) transitions.5d, 5e, 5f The nature of the electronic transitions were assigned by means of time dependent density functional theory calculations (TD‐DFT),^19^ which indicates that, in all cases, the vertical transition leading to the lowest energy absorptions involves the one‐electron promotion from a π‐molecular orbital localized on the carbazole moiety to π* orbitals localized on the carbene ligands, that is π–π* transitions (Supporting Information, Table S1 and Figure S36). For the T‐shaped complexes, the absorption energies follow the trend Cu > Au > Ag, which does not correspond to the trend of the extinction coefficients of the metal complexes, nor does it follow the trend of stronger bonding of the metal favoring the carbene over the carbazolide ligand (illustrated by the ratio of C−M/M−N bond lengths decreasing in the order Cu (0.95)9c>Ag (0.92, see Figure 2)>Au (0.86)11f).5f

**Table 1:** Photophysical data of [H_3_CNC]PF_6_⋅Cl and complexes **1**–**5**.

Complex	Medium	*T* [K]	Electronic absorption *λ*_max_ [nm] (*ϵ* [dm^3^ mol^−1^ cm^−1^])	Emission *λ*_em_ [nm] (*τ*_o_ [μs])	*Φ*_PL_^[b]^
**1**	THF^[*a*]^	298	334 (20,780), 367 (13,500), 402 (12,560), 464 (2,310)	460 (<0.1)	0.008
	butyronitrile glass	77		440, 560 (1720)	
	2‐MeTHF glass	77		432, 455, 487 (0.1)	
**2**	THF^[*a*]^	298	265 (52,100), 320 (12,200), 337 (11,880), 372 (8,040), 451 (8,820), 506 (7,520)	640 (2.0)	0.024
	acetonitrile	298	258 (53,190), 362 (10,800), 439 (8310), 502 (6,600)	654 (0.4)	0.003
	butyronitrile glass	77		590, 634 (4750)	
	2‐MeTHF glass	77		595, 642, 703 (3440)	
**3**	THF^[*a*]^	298	270 (48,950), 326 (15,480), 373 (10,480), 443 (8,360), 498 (8,150)	510, 550, 592, 652 (4.6)	0.006
	acetonitrile	298	239 (48,740), 261 (41,160), 372 (9,050), 417 (5,570), 489 (3,490)	527 (0.4)	0.003
	butyronitrile glass	77		492, 530, 569 (2410)	
	2‐MeTHF glass	77		492, 523, 560 (0.3)	
**4**	THF^[*a*]^	298	264 (46,250), 333 (9,140), 366 (6,280), 399 (5,530)	545 (170)	0.14
	acetonitrile	298	247 (58,540), 263 (50,790), 332 (10,660), 364 (7,520), 397 (5,940)	552 (51.4)	0.061
	butyronitrile glass	77		528 (810)	
	2‐MeTHF glass	77		505, 536, 579 (616)	
**5**	THF^[*a*]^	298	265 (31,080), 302 (7,420) 346 (5,460), 372 (6,480), 405 (5,340)	506, 542, 590 (6.7)	0.009
	butyronitrile glass	77		491, 531, 575 (2000)	
	2‐MeTHF glass	77		491, 529, 570, 617 (0.1)	
**[H_3_CNC]PF_6_⋅Cl**	THF^[*a*]^	298	294 (11,140), 333 (9,780), 378 (7,630)	500	0.020
	butyronitrile glass	77		500	
	2‐MeTHF glass	77		433, 481, 512, 554

[a] Measured at concentration =1×10^−5^  M. [b] The relative luminescence quantum yield was measured by the optical dilute method with a degassed aqueous solution of quinine sulfate in 1.0 N sulfuric acid (*Φ*=0.546, excitation wavelength at 365 nm) that was used as the reference.

**Figure 4:**
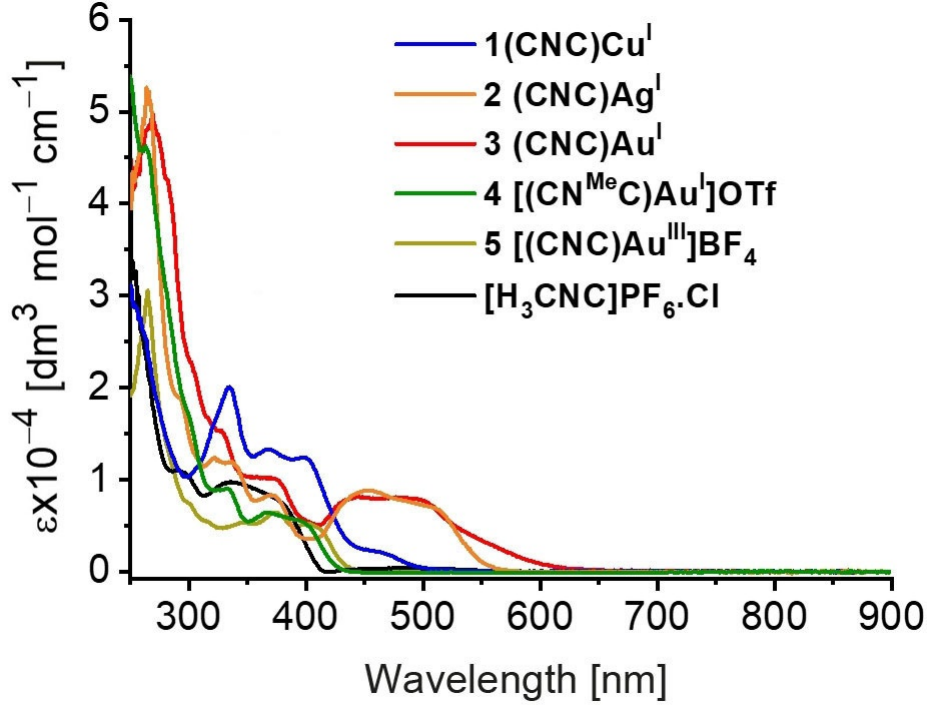
Electronic absorption spectra of ligand precursor and complexes **1**–**5** in THF at 298 K.

**Figure 5:**
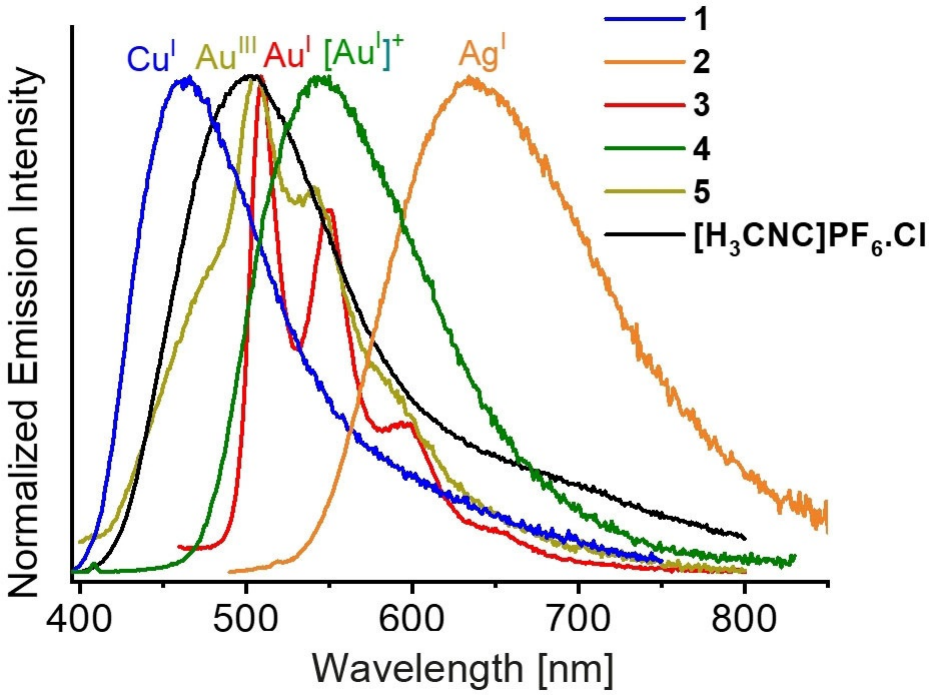
Normalized emission spectra of ligand precursors and complexes **1**–**5** in degassed THF at 298 K.

**Figure 6:**
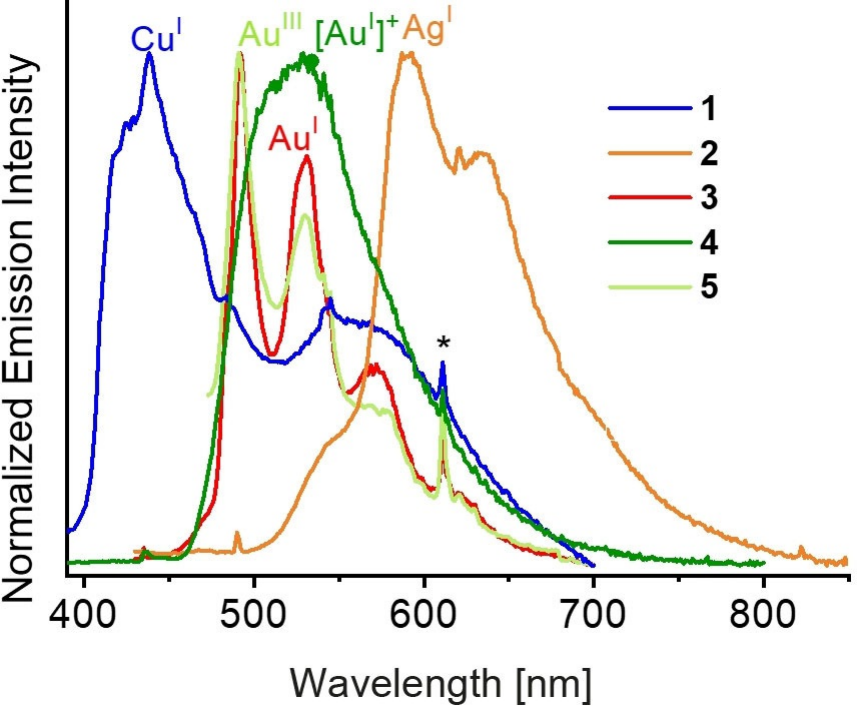
Normalized emission spectra of **1**–**5** in butyronitrile glass at 77 K; the asterisk represents an instrumental artifact.

In all cases, the TD‐DFT calculations indicate that the most stable excited state of the complexes is a triplet (T_1_), with the first singlet S_1_ resting above, but close to T_1_, which is compatible with an efficient S_1_–T_1_ ISC (ISC = intersystem crossing).^19^ The computed spin densities (Figure S37, Supporting Information) on the optimized structures of these triplet states indicate that the unpaired electrons in the T_1_ states are mainly located on the M−N bond, regardless of the nature of the transition metal. The lowest contribution of the metal is found for Cu^I^, in agreement with the significantly lower extinction coefficient found for **1**, compared to **2** and **3**.

The emission band of the ligand precursor in THF at 298 K is broad and featureless, which is typical of emissions of [π(carbazole)→π*(triazolium)] ILCT origin. The emission spectra recorded for complexes **1**, **2** and **4** show similar band shape to that of the ligand precursor, and therefore the emission bands are tentatively assigned as originating from metal‐perturbed [π(carbazolide)→π*(carbene)] ^3^ILCT excited state.

The emission of the two‐coordinated complex **4** is red‐shifted when compared to the ligand precursor, owing to the coordination of Au^I^ metal centre to the carbene moieties. On the other hand, complex **5** showed a vibronic emission band, which is tentatively assigned as originating from an intraligand (^3^IL) excited state, probably arising from the higher oxidation state of the Au^III^ centre that reduces the ease of donation from the carbazolide, leading to a higher‐lying ^3^ILCT state. For complex **3**, vibronic‐structured ^3^IL emission is observed in THF, whereas structureless ^3^ILCT emission is found in acetonitrile, showing that the ^3^ILCT and ^3^IL excited states are close in energy, and their energies are affected by solvent polarity. The observation of the structureless emission can be attributed to the stabilization of the ^3^ILCT state by the polar acetonitrile, which leads to the ^3^ILCT state becoming the lowest energy excited state responsible for emission. In glass matrices at 77 K, the complexes in general showed vibronic emission bands, originating from the ^3^IL excited state, probably due to the destabilization of the ^3^ILCT state in glass matrices. The photoluminescence quantum yields of the compounds are low, ranging from 0.6 % for **3** (T‐shaped Au^I^) to 14 % for **4** (linear Au^I^). The emission wavelengths range over 180 nm (≈6100 cm^−1^) with the use of different metals for color‐tuning in the T‐shaped complexes^20^ (**1** Cu^I^ *λ*_em_=460; **3** Au^I^ *λ*_em_=510–652 nm; **2** Ag^I^ *λ*_em_=640 nm), and the copper(I) complex displaying uncommon blue emission.7c, 21

The luminescence lifetimes of the complexes were measured at 298 K and 77 K. The lifetime of complex **1** is too short to be determined with certainty in THF at 298 K, and the emission may be fluorescent in nature.^22^ In acetonitrile, for complexes **2**–**4**, the decay lifetimes are in the microsecond range for **2** (*τ*_0_=0.4 μs), **3** (*τ*_0_=0.4 μs) at room temperature, but increase to *τ*_0_=4.75 ms and 2.41 ms, respectively, upon cooling to 77 K in butyronitrile glass. The increase in excited state lifetime is in line with the assignment of a change in emission origin from predominantly ^3^ILCT to ^3^IL excited state, the latter of which typically shows longer lifetime. Such increase in *τ*_0_ at lower temperatures by four orders of magnitude may also suggest the occurrence of TADF, with thermal population of S_1_ allowed due to fast reversible or equilibrium ISC (rISC) if Δ*E*(T_1_−S_1_) <0.37 eV (Table S2, Supporting Information).1b, 6a, 7c, 23 This corresponds to the known efficiency trade‐off in which smaller Δ*E*(T_1_−S_1_) leads to lower radiative rates and *Φ*_PL_.^6^ However, to confirm the occurrence of TADF, temperature‐dependent lifetime measurements at more data points would be required to determine the contribution of radiative and non‐radiative decay rates.

Comparing the gold complexes **3**–**5**, the calculated energy transitions (both Δ*E*(T_1_−S_0_) and Δ*E*(T_1_−S_1_) (Table S2)), match that of the experimental emission energies, with **3**<**5**<**4** (Table S2). In this case, all three gold complexes have green emissions. For the linear Au^I^ complex **4**, Δ*E*(T_1_−S_1_)=0.419 eV, which is prohibitive for rISC (T_1_→S_1_) and should suppress TADF. Greater phosphorescence contribution to the photoluminescence leads to longer decay times in THF at 298 K (*τ*_0_=170 μs for **4**, compared to *τ*_0_=4.6 and 6.7 μs, for **3** and **5**, respectively). A significantly lesser increase in decay time for **4** upon cooling to 77 K in butyronitrile glass (*τ*_0_=810 μs, compared to *τ*_0_=2410 and 2000 μs, for **3** and **5**, respectively), together with the highest *Φ*_PL_ observed for **4**, supports such increased phosphorescence contribution. In conclusion, the bis(mesoionic carbene)carbazolide CNC pincer ligand allows for modulation of the photophysical properties of complexes, either with the introduction of different metals to access emissions from the blue to orange spectrum (*d* ^10^ metals **1**–**3**), or by modification of the complex with electrophilic attack at either the carbazolide‐N (**4**) or at the nucleophilic metal centre (**5**).

## Experimental Section

The precursor **[H_3_CNC]PF_6_⋅Cl** and complexes **1**,9c **3** 11f and **4** 11f were prepared following literature procedures, whereas the synthesis and characterization of **2** and **5** are given in the Supporting Information for this paper. The molecular structures of **2** and **5** were determined by single crystal X‐ray crystallography.^24^ The details of the data collection and structure solution are given for each compound in the Supporting Information.

## Conflict of interest

The authors declare no conflict of interest.

## Supporting information

As a service to our authors and readers, this journal provides supporting information supplied by the authors. Such materials are peer reviewed and may be re‐organized for online delivery, but are not copy‐edited or typeset. Technical support issues arising from supporting information (other than missing files) should be addressed to the authors.

Supplementary
