# Supplementary material for: Synthesis and Photophysical Properties of T‐Shaped Coinage‐Metal Complexes
Source: Chemistry. 2020 May 15;26(31):6993–8. doi: 10.1002/chem.202000726 (PMC7317956; doi:10.1002/chem.202000726)
Supplement: Supplementary file 1 — Supplementary [file CHEM-26-6993-s001.pdf]

Chemistry–A European Journal

Supporting Information

Synthesis and Photophysical Properties of T-Shaped Coinage-Metal Complexes

George Kleinhans,[a, b] Alan K.-W. Chan,[c] Ming-Yi Leung,[c] David C. Liles,[a] Manuel A. Fernandes,[b] Vivian W.-W. Yam,[c] Israel Fernández,*[d] and Daniela I. Bezuidenhout*[b, e]

Table of Contents

Section S1: Method, Materials and Characterization Techniques 2
a) Method 2
b) Materials 2
c) Characterization Techniques 2
i. Nuclear Magnetic Resonance Spectroscopy 2
ii. Mass Spectrometry 3
iii. Cyclic Voltammetry 4
Section S2: Synthesis details and characterization 5
a) Synthesis of (CNC)Ag^{I} (**2**) 5
b) Synthesis of [(CNC)Au^{III}F]BF_4 (**5**) 5
Section S3: NMR Spectra of Selected Complexes 7
Section S4: Single Crystal X-ray Diffraction 9
Section S5: Absorption, emission spectra and PLQY measurements 10
Section S6: Computational Details 25
Section S7: References 61

Section S1: Method, Materials and Characterization Techniques

a) Method

All synthetic manipulations, unless otherwise stated, were performed under an N_2 or Ar atmosphere using oven or flame dried glassware and standard Schlenk or vacuum line techniques. Air sensitive solids where stored and handled in a PureLab HE glove box. Preparation of NMR and crystallization samples that also require an inert atmosphere were performed in a glove box.

b) Materials

The ligand precursor $[H_3CNC]PF_6.Cl$ and (CNC)Cu^I complex **1**, was prepared as previously reported by our group.[1] The gold complexes **3** and **4** were also prepared as reported by our group.[2] All other reagents were obtained from commercial sources and were used without any further purification.

Unless otherwise stated, only anhydrous solvents were used for the experimental procedures. Anhydrous THF and Et_2O were obtained after distillation over sodium and benzophenone under a N_2 atmosphere. Anhydrous toluene and hexane were obtained after distillation over sodium under a N_2 atmosphere. Anhydrous CH_2Cl_2 was obtained after distillation over calcium hydride under a N_2 atmosphere.

c) Characterization Techniques

i. Nuclear Magnetic Resonance Spectroscopy

Nuclear magnetic resonance (NMR) spectra were obtained using either a Bruker AVANCE-III-300 operating at 300.13 MHz for ^{1}H, 75.47 MHz for ^{13}C, 121.49 MHz for ^{31}P and 282.40 MHz for ^{19}F; or AVANCE-III-400 operating at 400.21 MHz for ^{1}H, 100.64 MHz for ^{13}C, 162.01 MHz for ^{31}P and 376.57 MHz for ^{19}F. ^{1}H Chemical shifts are reported as δ (ppm) values downfield from Me_4Si and chemical shifts where referenced to residual non-deuterated solvent peaks ($CDCl_3$, 7.26 ppm; CD_2Cl_2, 5.32 ppm). ^{13}C chemical shifts are also reported as δ (ppm) values downfield from Me_4Si and chemical shifts were referenced to residual non-deuterated solvents peaks ($CDCl_3$, 77.16 ppm; CD_2Cl_2, 54.00 ppm). Proton coupling constants (*J*) are given in Hz. The spectral coupling patterns are designated as follows: s/S - singlet; d/D - doublet; t/T - triplet; q/Q - quartet; sept - septet; hept - heptet; m - multiplet; br - broad signal. Quaternary carbons are designated as $\mathbf{C}_q$.

Chemical shift assignments in the ^{1}H NMR spectra are based on first-order analysis and when required were confirmed by two-dimensional (2D) (^{1}H-^{1}H) homonuclear chemical shift correlation (COSY) experiments. The ^{13}C shifts were obtained from proton-decoupled ^{13}C NMR spectra. Where necessary, the multiplicities of the ^{13}C signals were deduced from proton-decoupled DEPT-135 spectra. The resonances

of the proton-bearing carbon atoms were correlated with specific proton resonances using 2D (^{13}C-^{1}H) heteronuclear single-quantum coherence (HSQC) and heteronuclear multiple bond correlations (HMBC) experiments. Standard Bruker pulse programs were used in the experiments.

ii. Mass Spectrometry

Mass Spectral Analysis of (CNC)Ag^{I} complex ***2***

Chemicals and reagents: MS-grade acetonitrile was purchased from Romil. Acetonitrile with 0.1% formic acid was purchased from Fluka.

Flow Injection Analysis (FIA)-Electrospray ionization (ESI)-Mass spectrometry (MS): ESI mass spectra were acquired in both positive and negative ionization mode using a Waters, Synapt G2 mass spectrometer (Milford, MA, USA). Prior to analysis, a 5 mM sodium formate solution was used to calibrate the instrument in resolution mode achieving a mass accuracy of less than 0.5 mDa over a mass range of 100-1200 Da using the IntelliStart function of Masslynx software. For analysis, the instrument was operated under the following conditions: capillary voltage 2.8 kV (positive mode) or 2.5 kV (negative mode); sampling cone (ramped from 20 V - 40 V), extraction cone 4 V, source temperature 100°C, desolvation temperature 200°C, Cone gas 100 L/h, desolvation gas 500 L/h, MS gas: nitrogen.

The sample was made up in 100% acetonitrile (MeCN) to an approximate concentration of 10 µg/ml. Flow injection using MeCN (0.1% formic acid) as mobile phase (with the aid of an Acquity autosampler injecting 5 µl of sample) was used to introduce the compounds to the ionization source such that a minimum of 10 scans with a scan time of 0.5 seconds in continuum format could be combined across a bolus peak. A 2 ng/µl solution of Leucine enkephalin was constantly infused via a syringe through a separate ESI probe with an angle perpendicular to the sample spray at a flow rate of 3 µL/min and was sampled every 10 seconds to compensate for possible experimental drift. The mass spectral resolution attained was typically > 15000 (FWHM definition) as calculated using ResCalc (version 2.2.3). Quality control (QC) samples consisted of sodium formate clusters that were likewise analysed and used to confirm that the method was working. The attained spectrum list was saved as a txt file and further processed using mMass.

Mass Spectral Analysis of [(CNC)Au^{III}F]BF_4 complex ***5***

Electrospray mass spectra (ESI-MS) were recorded on a Micromass Quatro LC instrument or on a Bruker QTOF Mass spectrometer with positive electron spray as the ionization techniques; nitrogen was employed as drying and nebulizing gas at a flow of 4 L/min. The m/z values were measured in the range of 100–1500 with acetonitrile as solvent. Accurate mass measurements were performed by use of a Q-

TOF premier mass spectrometer with electrospray source (Waters, Manchester, UK) operating at a resolution of ca. 16000 (fwhm).

iii. Cyclic Voltammetry

Electrochemical studies were carried out using Metrohm µAutolab type III potentiostat linked to a computer using GPES Electrochemistry software, in conjunction with a three-electrode cell. The working electrode was a glassy carbon disc (3.0 mm diameter) and the counter electrode was a platinum wire. The reference was a non-aqueous Ag/Ag^{+} electrode separated from the test solution by a fine porosity frit. Solutions in THF were 1.0×10^{-3} mol dm^{-3} in the test compound and internal references $[Cp_2{*}Fe]^{0/+1}$ and $[Cp_2Fe]^{0/+1}$, and 0.1 mol dm^{-3} in $[^{n}Bu_4N][PF_6]$ as the supporting electrolyte. All E_p^{ox}, E_p^{red} and $E^{0\prime}$ values are at scan rates of 100 mV s^{-1}.

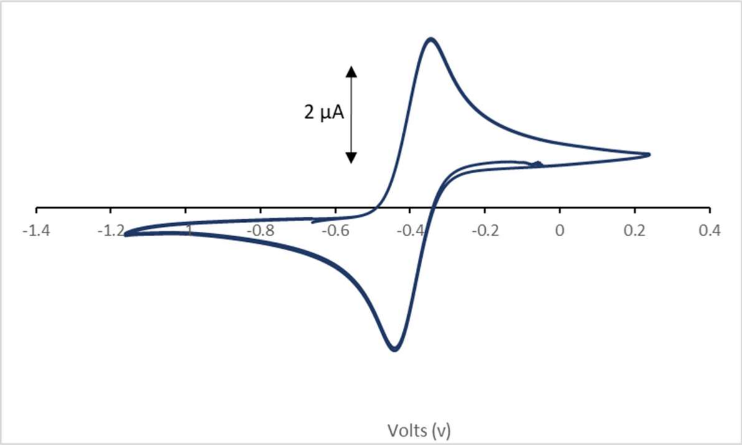

Figure S1. The cyclic voltammogram of **3**, at a glassy carbon electrode, scan rate 0.1 V s^{-1} in THF (Internal standard $[Cp_2Fe]^{0/+1}$ not indicated).

Section S2: Synthesis details and characterization

a) Synthesis of (CNC)Ag^{I} (**2**)

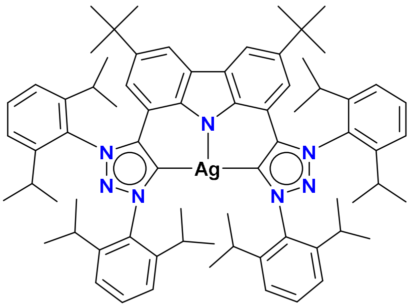

The silver complex was prepared, through an adaptation of the procedure reported by Crudden and co-workers.[3] A flame dried Schlenk was charged with **[H_3CNC]PF_6·Cl** (1.00 g, 8.1 x 10^{-4} mol), Ag_2O (749.4 mg, 3.2 x 10^{-3} mol), and KBr (962.0 mg, 8.1 x 10^{-3} mol). The reaction vessel was evacuated and purged with N_2 (g), followed by addition of DCM (25 mL). The solution was stirred at room temperature for five days, in the absence of light. The solvent was evaporated *in vacuo*. The product was extracted from the residue with Et_2O (6 x 25 mL), followed by *in vacuo* evaporation of the solvent. The residue was washed with hexane (5 x 20 mL), followed by extraction of the product with toluene (4 x 20 mL). Evaporation of the solvent *in vacuo*, yielded **2** (295.0 mg, 2.5 x 10^{-4} mol, 31%) as a dark orange solid. Crystallisation from DCM yielded single crystals suitable for XRD analysis. **^{1}H NMR** δ_H ($CDCl_3$, 300 MHz) 8.17 (broad s, 2H, Ar**H**$_{carb}$), 7.63 (t, *J* = 7.8 Hz, 2H, Ar**H**$_{Dipp}$), 7.47 (d, *J* = 7.8 Hz, 4H, Ar**H**$_{Dipp}$), 7.45 (m, 2H, Ar**H**$_{Dipp}$ overlaps with Ar**H**$_{Dipp}$), 7.25 (broad s, 2H, Ar**H**$_{carb}$), 7.14 (d, *J* = 7.8 Hz, 4H, Ar**H**$_{Dipp}$), 2.71 – 2.53 (m, 8H, C**H**(CH_3)$_2$), 1.12 (d, *J* = 6.9 Hz, 12H, CH(C**H**$_3$)$_2$), 1.07 (d, 12H, CH(C**H**$_3$)$_2$ overlaps with C(CH_3)$_3$), 1.06 (s, 18H, C(C**H**$_3$)$_3$), 1.02 (d, *J* = 7.2 Hz, 12H, CH(C**H**$_3$)$_2$), 0.99 (d, *J* = 7.2 Hz, 12H, CH(C**H**$_3$)$_2$). **^{13}C NMR** δ_C ($CDCl_3$, 75 MHz) 177.4 (dd, *J* = 185.3 Hz, 13.2 Hz, Ag-**C**$_{Carbene}$), 148.7 (Ar**C**$_q$), 148.6 (Ar**C**$_q$), 146.0 (Ar**C**$_q$), 145.5 (Ar**C**$_q$), 145.0 (Ar**C**$_q$), 138.0 (Ar**C**$_q$), 135.8 (Ar**C**$_q$), 135.7 (Ar**C**$_q$), 131.3 (Ar**C**H), 129.7 (Ar**C**H), 126.7 (Ar**C**$_q$), 125.4 (Ar**C**H), 123.6 (Ar**C**H), 120.3 (Ar**C**H), 117.9 (Ar**C**H), 111.5 (Ar**C**$_q$), 34.4 (**C**(CH_3)$_3$), 32.1 (C(**C**H$_3$)$_3$), 28.9 (**C**H(CH_3)$_2$), 28.6 (**C**H(CH_3)$_2$), 25.4 (CH(**C**H$_3$)$_2$), 24.7 (CH(**C**H$_3$)$_2$), 24.2 (CH(**C**H$_3$)$_2$), 23.3 (CH(**C**H$_3$)$_2$). **HRMS** (FIA-ESI): Calculated for $C_{72}H_{90}N_7Ag^{2+}$ $[M + 2H]^{2+}$: 580.8227, found: 580.8235.

b) Synthesis of [(CNC)Au^{III}F]BF_4 (**5**)

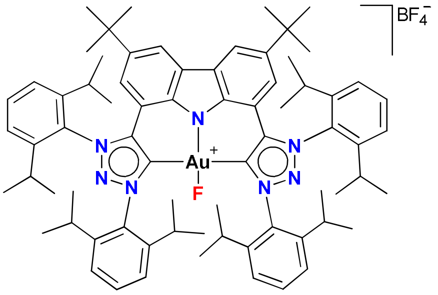

A Schlenk tube was loaded with **(CNC)Au^{I}** (120.0 mg, 9.6 x 10^{-5} mol), Selectfluor (35.7 mg, 1.0 x 10^{-4} mol), evacuated and purged with Ar (g). The solids were cooled down to -78 °C. To the vessel was added THF (15 mL) which was also cooled down to -78 °C. The reaction was stirred at this temperature for 10 minutes, before being gradually warmed up to room temperature whilst stirring in the absence of light. The solution was stirred overnight. The solution was filtered and the solvent evaporated under reduced pressure. The residue was washed with toluene (4 x 10 mL) and then with Et_2O

(4 x 10 mL). The product was extracted with a mixture of CH_2Cl_2 and toluene in a 1:2 ratio (2 x 10 mL), respectively. The solvent was evaporated under reduced pressure. The residue was washed with hexane (4 x 10 mL) and the solid was dried *in vacuo*, to yield **5** (48.0 mg, 3.5 x 10^{-5} mol, 37%) as a light green solid. Crystals suitable for X-ray diffraction analysis were obtained through slow evaporation of a CD_2Cl_2 solution of **5**. **^{1}H NMR** δ_H (CD_2Cl_2, 400 MHz) 8.37 (d, *J* = 1.6 Hz, 2H, Ar$\mathbf{H}_{carb}$), 7.85 (t, *J* = 8.0 Hz, 2H, Ar$\mathbf{H}_{Dipp}$), 7.62 (d, *J* = 8.0 Hz, 4H, Ar$\mathbf{H}_{Dipp}$), 7.49 (t, *J* = 8.0 Hz, 2H, Ar$\mathbf{H}_{Dipp}$), 7.28 (d, *J* = 1.6 Hz, 2H, Ar$\mathbf{H}_{carb}$), 7.17 (d, *J* = 8.0 Hz, 4H, Ar$\mathbf{H}_{Dipp}$), 2.33 (sept, *J* = 6.8 Hz, 4H, C**H**(CH_3)$_2$), 2.23 (sept, *J* = 6.8 Hz, 4H, C**H**(CH_3)$_2$), 1.20 (d, *J* = 6.8 Hz, 12H, CH(C**H**$_3$)$_2$), 1.13 (s, 18H, C(C**H**$_3$)$_3$), 1.11 (d, *J* = 7.2 Hz, 12H, CH(C**H**$_3$)$_2$ overlaps with CH(CH_3)$_2$), 1.09 (d, *J* = 7.8 Hz, 12H, CH(C**H**$_3$)$_2$ overlaps with CH(CH_3)$_2$), 1.03 (d, *J* = 6.8 Hz, 12H, CH(C**H**$_3$)$_2$). **^{13}C NMR** δ_C (CD_2Cl_2, 100 MHz) n.o. ($\mathbf{C}_{carbene}$), 146.0 (Ar$\mathbf{C}_q$), 145.0 (Ar$\mathbf{C}_q$), 144.9 (Ar$\mathbf{C}_q$), 143.8 (Ar$\mathbf{C}_q$), 141.9 (Ar$\mathbf{C}_q$), 138.1 (Ar$\mathbf{C}_q$), 136.5 (Ar$\mathbf{C}_q$), 134.6 (Ar$\mathbf{C}_q$), 134.5 (Ar$\mathbf{C}_q$), 134.0 (Ar**C**H), 132.7 (Ar$\mathbf{C}_q$), 131.6 (Ar**C**H), 126.7 (Ar**C**H), 126.0 (Ar$\mathbf{C}_q$), 124.0 (Ar**C**H), 121.1 (Ar**C**H), 120.5 (Ar**C**H), 108.3 (Ar$\mathbf{C}_q$), 35.0 (**C**(CH_3)$_3$), 31.8 (C(**C**H$_3$)$_3$), 29.9 (**C**H(CH_3)$_2$), 25.4 (CH(**C**H$_3$)$_2$), 25.1 (CH(**C**H$_3$)$_2$), 24.4 (CH(**C**H$_3$)$_2$), 23.6 (CH(**C**H$_3$)$_2$). **^{19}F NMR** δ_F (CD_2Cl_2, 282 MHz) -299.9 (Au**F**), -153.2 (B**F**$_4$). **HRMS** (ESI-TOF): Calculated for $C_{72}H_{90}N_7AuF^+$ [M]$^+$: 1268.6907, found: 1268.6650.

Section S3: NMR Spectra of Selected Complexes

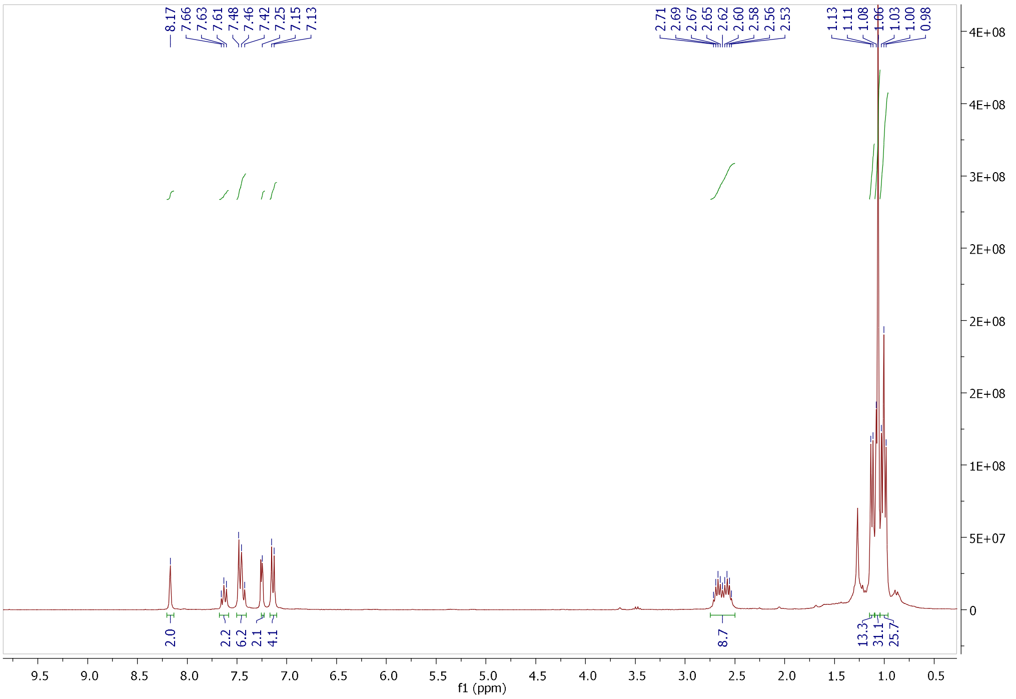

Figure S2: ^{1}H NMR spectrum of **2** in $CDCl_3$

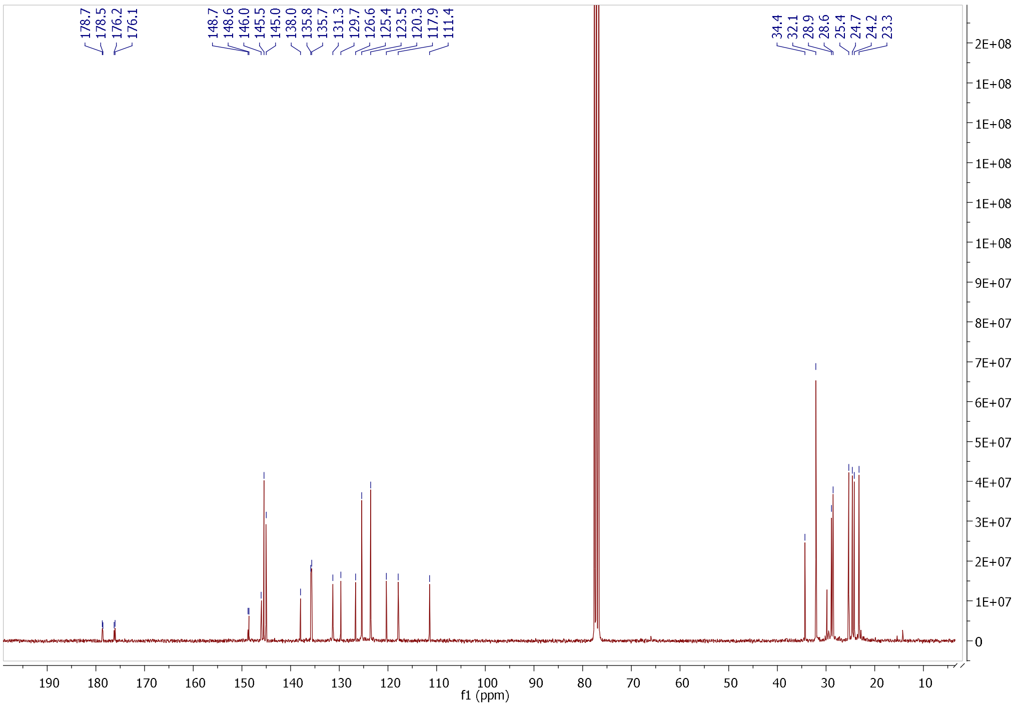

Figure S3: ^{13}C NMR spectrum of **2** in $CDCl_3$

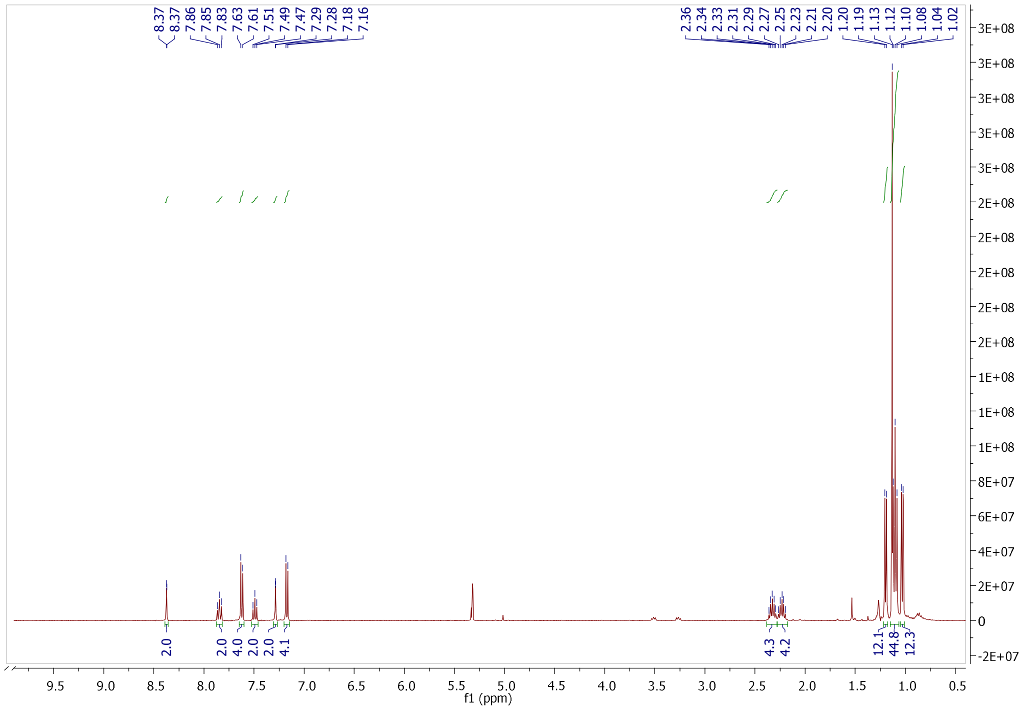

Figure S4: ^{1}H NMR spectrum of **5** in CD_2Cl_2

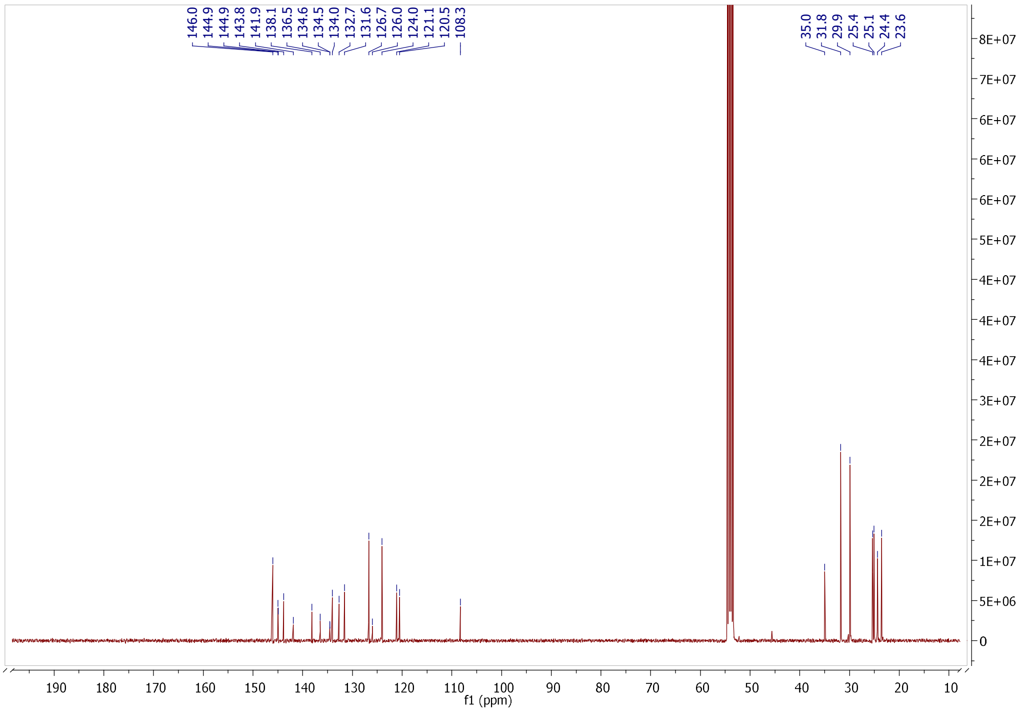

Figure S5: ^{13}C NMR spectrum of **5** in CD_2Cl_2

Section S4: Single Crystal X-ray Diffraction

Single crystal X-ray diffraction data were collected on a Bruker D8 Venture kappa geometry diffractometer using I µS micro source Mo-Kα radiation (λ = 0.71073 Å), a Photon 100 CMOS detector and Apex2 control software.[4] Data reductions were performed using SAINT[4] and multi-scan absorption corrections were applied using SADABS.[4] The structures were solved using the Bruker-SHELXTS structure solution program using dual-space methods.[5]

*(CNC)Ag^{I} complex **2***

A single crystal was mounted on a needle, and then placed in a cold stream of N_2 at 150 K. The structure was refined using both Bruker-SHELXTL[6] and SHELXL-2017/1.[6]

Crystal data for 2: $C_{74}H_{94}AgCl_4N_7$ (*M* = 1331.23 g/mol): triclinic, space group *P-1* (no. 2), *a* = 14.8607(5) Å, *b* = 15.7212(6) Å, *c* = 18.0365(6) Å, *α* = 85.1075(14)°, *ß* = 69.7219(13)°, *γ* = 63.4722(13)°, *V* = 3524.4(2) Å^3, *Z* = 2, *T* = 150(2) K, µ(MoKα) = 0.483 mm^{-1}, *Dcalc* = 1.254 g/cm^3, 175814 reflections measured (4.558° ≤ 2Θ ≤ 61.02°), 21515 unique (R_{int} = 0.0318, R_{sigma} = 0.0188) which were used in all calculations. The final R_1 was 0.0348 (I > 2s(I)) and wR_2 was 0.0932 (all data).

*[(CNC)Au^{III}F]BF_4 complex **5***

A single crystal was selected under oil, mounted on a nylon loop and then immediately placed in a cold stream of N_2 at 173 K. The Structure was refined using both Bruker-SHELXTL[6] and OLEX2.[7]

Crystal Data for **5**: $C_{76}H_{98}AuBCl_8F_5N_7$ (*M* = 1704.03 g/mol): monoclinic, space group *C2/c* (no. 15), *a* = 25.550(2) Å, *b* = 23.099(2) Å, *c* = 15.3633(14) Å, β = 110.607(3), *V* = 8487.0(13) Å^3, *Z* = 4, *T* = 150(2) K, µ(MoKα) = 2.039 mm^{-1}, *Dcalc* = 1.334 g/cm^3, 146191 reflections measured (4.524° ≤ 2Θ ≤ 55.758°), 9970 unique (R_{int} = 0.0876, R_{sigma} = 0.0403) which were used in all calculations. The final R_1 was 0.0658 (I > 2σ(I)) and wR_2 was 0.1667 (all data).

For both structures, the contributions of highly disordered solvent molecules to the calculated structure factors [2($C_2H_2Cl_2$) in **2** and 4($C_2Cl_2D_2$) plus a small fraction (0.088) of the BF_4^- counter-ion in **5**] were modelled using PLATON SQUEEZE.[8]

Section S5: Absorption, emission spectra and PLQY measurements

UV/Vis spectra were obtained on a Hewlett–Packard 8452A diode-array spectrophotometer. Steady-state excitation and emission spectra at room temperature and at 77 K were recorded on an Edinburgh Instruments FS5 Spectrofluorometer equipped with a Hamamatsu R928P PMT detector. Samples for photophysical measurements were degassed with no less than four freeze–pump–thaw cycles on the high-vacuum line prior to measurements. Photophysical measurements in glass matrices at low temperature were carried out with the sample loaded in a quartz tube inside a quartz-walled Dewar flask filled with liquid nitrogen (77 K). Excited-state lifetimes of the glass and solution samples were measured by using a conventional laser system. The excitation source used was the λ=355 nm output (third harmonic, 8 ns) of a Spectra-Physics Quanta-Ray Qswitched GCR-150 pulsed Nd:YAG laser (10 Hz). Luminescence decay signals were detected by using a Hamamatsu R928 photomultiplier tube (PMT) and recorded on a Tektronix Model TDS- 620A (500 MHz, 2 GSs@1) digital oscilloscope. Luminescence quantum yields were measured by means of the optical dilute method reported by Demas and Crosby.[9,10] A degassed aqueous solution of quinine sulfate in 1.0 N sulfuric acid (F=0.546, excitation wavelength at λ=365 nm) was used as the reference and corrected for the refractive index of the solution.[10]

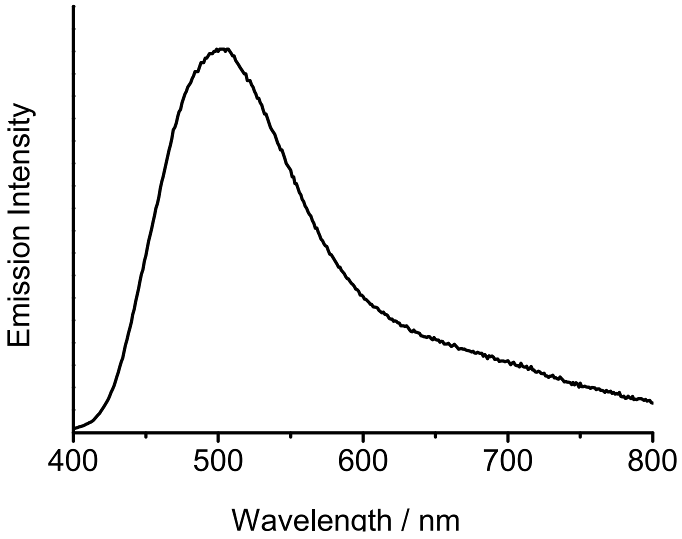

Figure S6: Emission spectrum of **[H_3CNC]PF_6.Cl** in degassed THF at 298 K

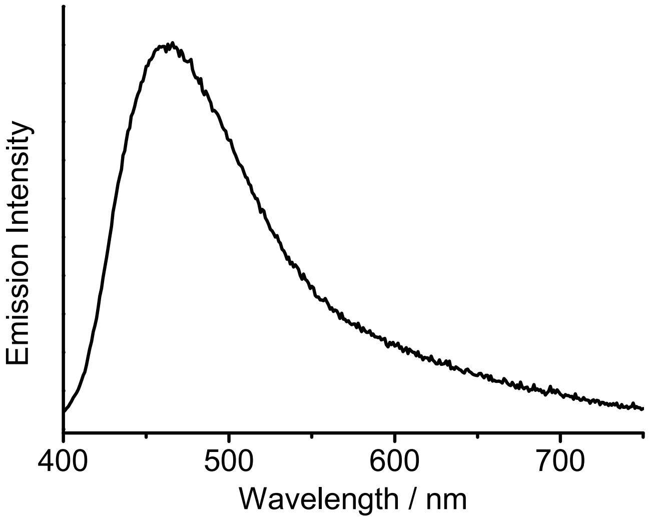

Figure S7: Emission spectrum of **1 (CNC)Cu**^{I} in degassed THF at 298 K

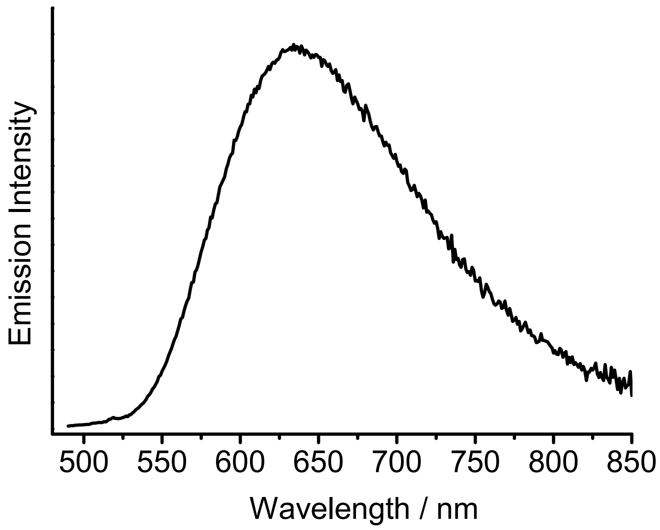

Figure S8: Emission spectrum of **2 (CNC)Ag**^{I} in degassed THF at 298 K

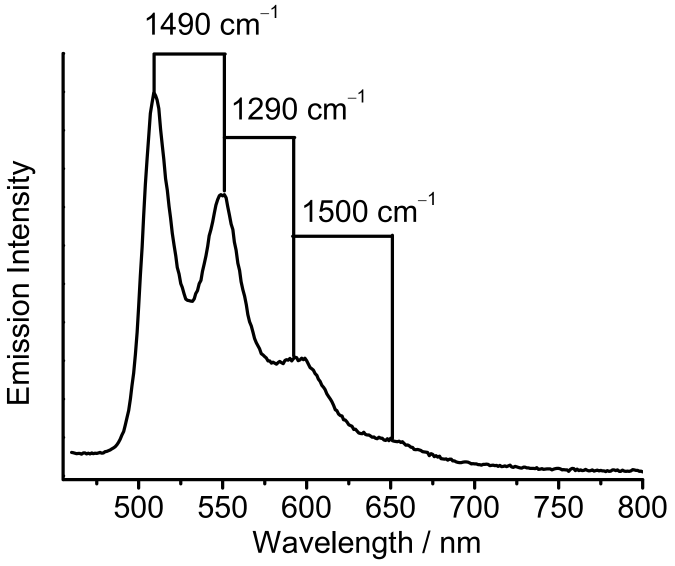

Figure S9: Emission spectrum of **3 (CNC)Au^{I}** in degassed THF at 298 K

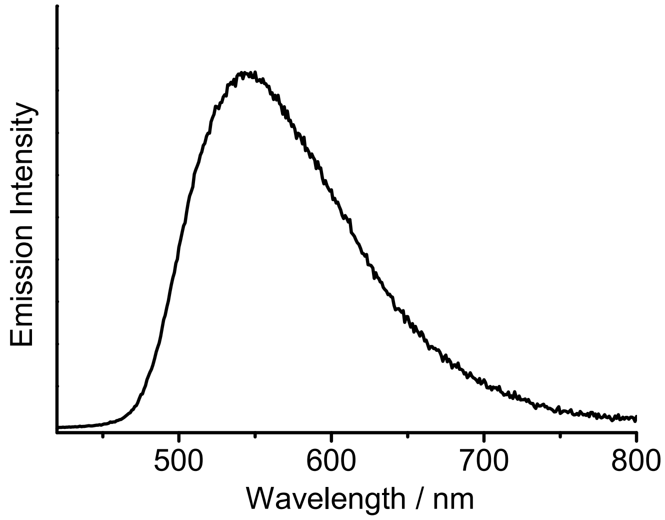

Figure S10: Emission spectrum of **4 [(CN^{Me}C)Au^{I}]OTf** in degassed THF at 298 K

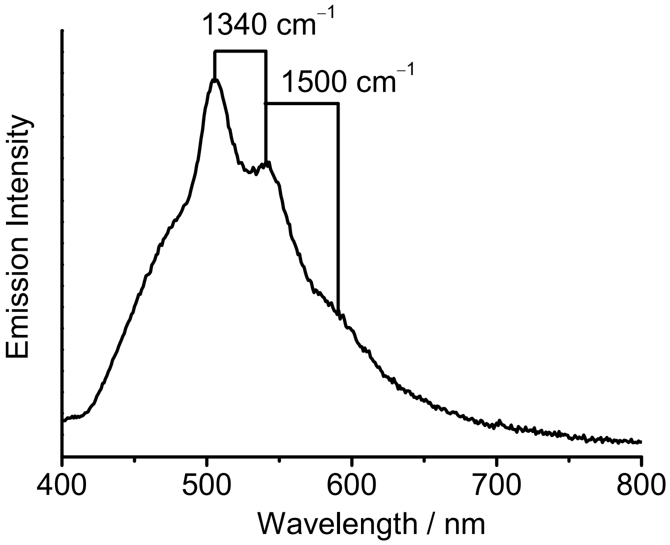

Figure S11: Emission spectrum of **5 [(CNC)Au^{III}]BF$_4$** in degassed THF at 298 K

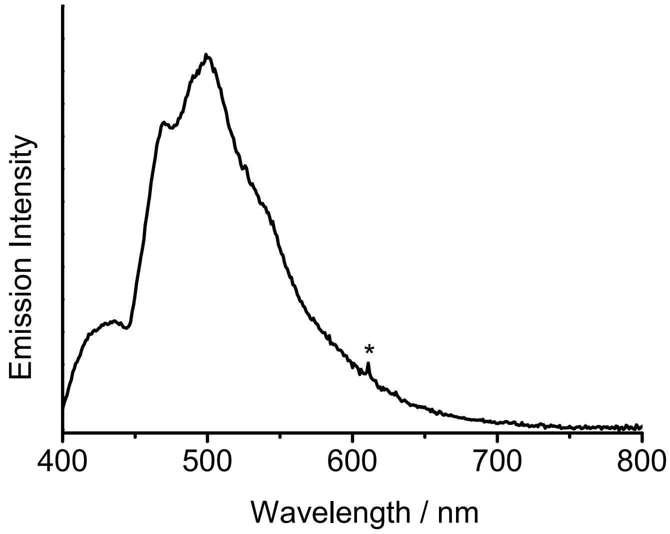

Figure S12: Emission spectrum of **[H$_3$CNC]PF$_6$.Cl** in butyronitrile glass at 77 K; * represents an instrumental artifact

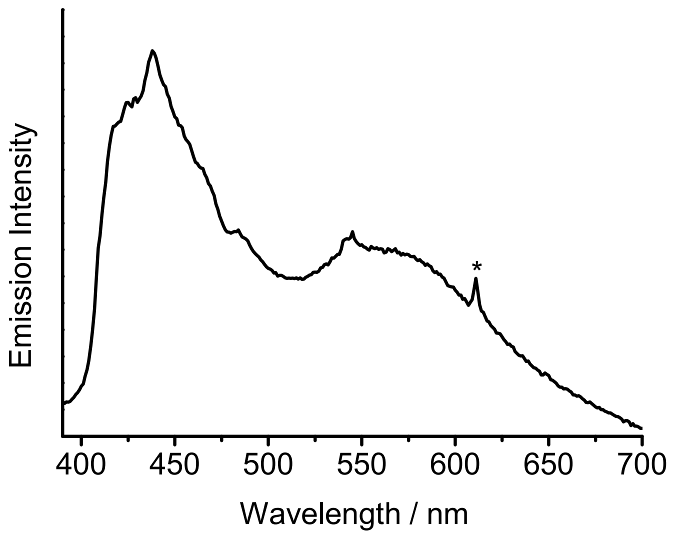

Figure S13: Emission spectrum of **1 (CNC)Cu**^{I} in butyronitrile glass at 77 K; * represents an instrumental artifact

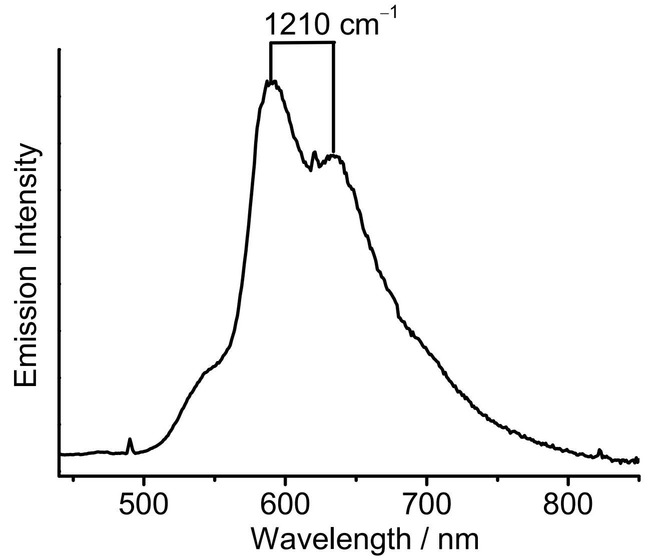

Figure S14: Emission spectrum of **2 (CNC)Ag**^{I} in butyronitrile glass at 77 K

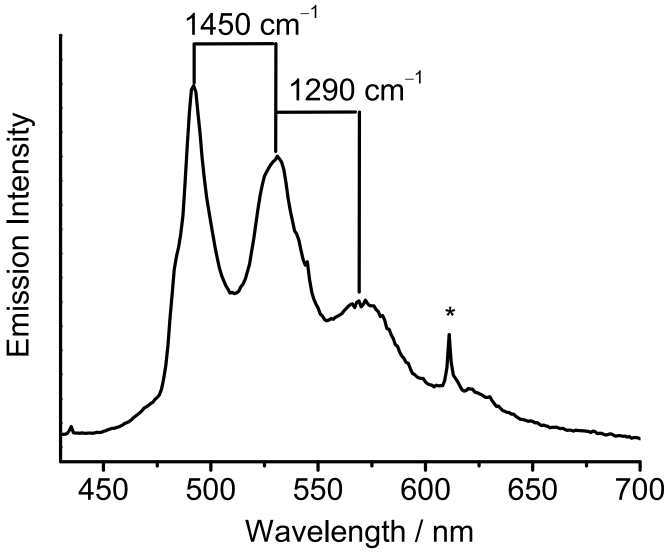

Figure S15: Emission spectrum of **3 (CNC)Au^{I}** in butyronitrile glass at 77 K; * represents an instrumental artifact

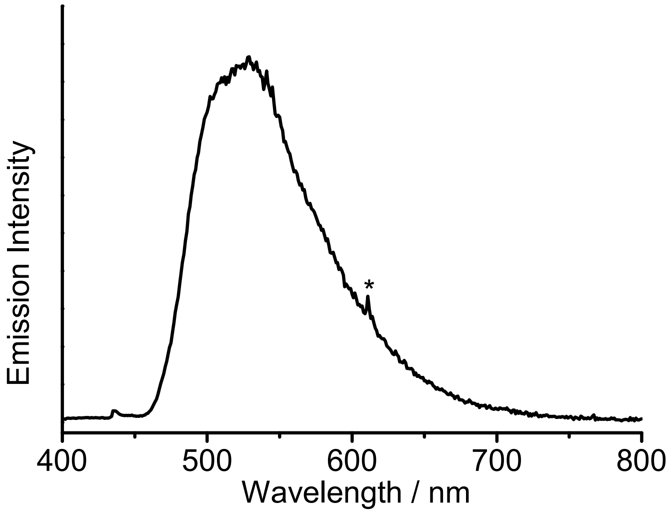

Figure S16: Emission spectrum of **4 [(CN^{Me}C)Au^{I}]OTf** in butyronitrile glass at 77 K; * represents an instrumental artifact

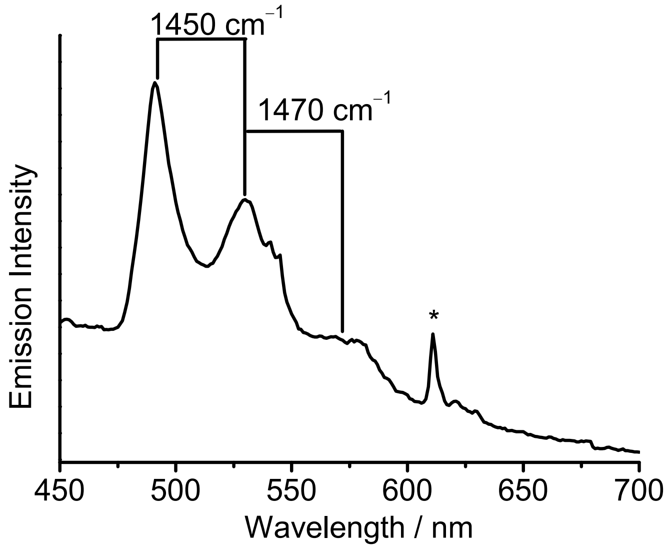

Figure S17: Emission spectrum of **5 [(CNC)Au^{III}]BF$_4$** in butyronitrile glass at 77 K; * represents an instrumental artifact

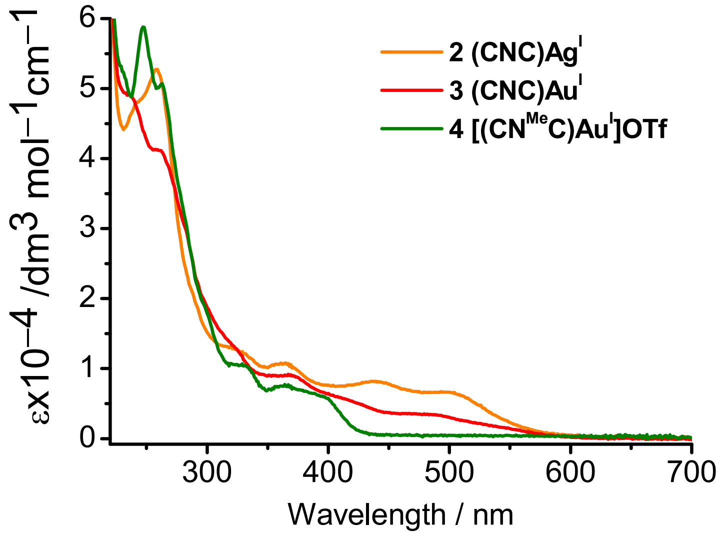

Figure S18. Electronic absorption spectra of complexes **2**–**4** in acetonitrile at 298 K

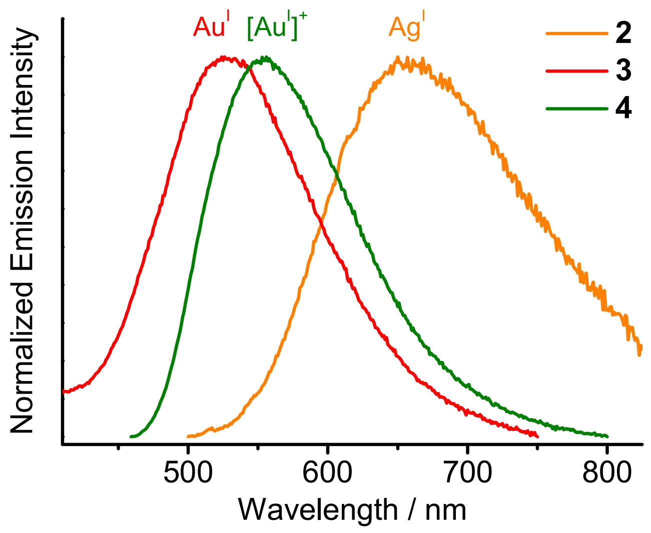

Figure S19: Normalized emission spectra of **2**–**4** in degassed acetonitrile at 298 K

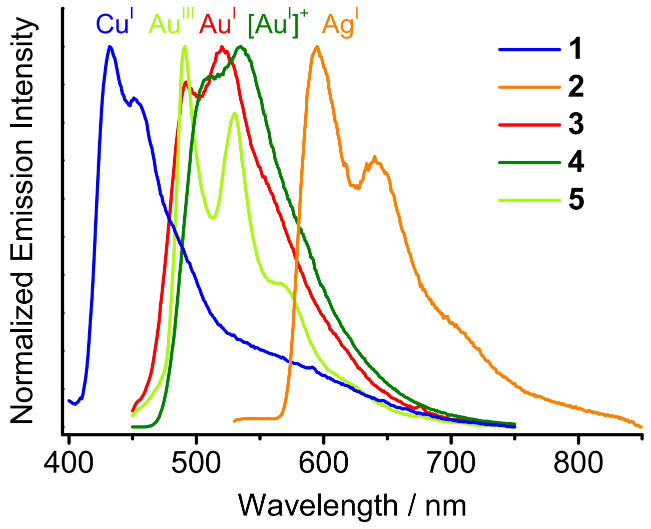

Figure S20: Normalized emission spectra of **1**–**5** in 2-methyltetrahydrofuran glass at 77 K

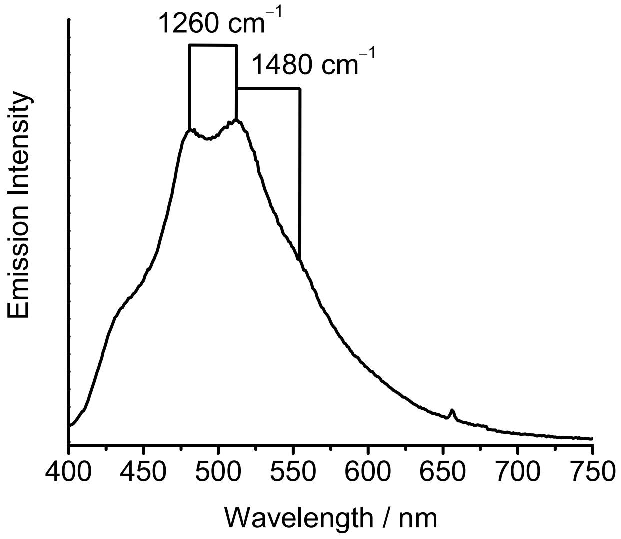

Figure S21: Emission spectrum of **[H_3CNC]PF_6.Cl** in in 2-methyltetrahydrofuran glass at 77 K

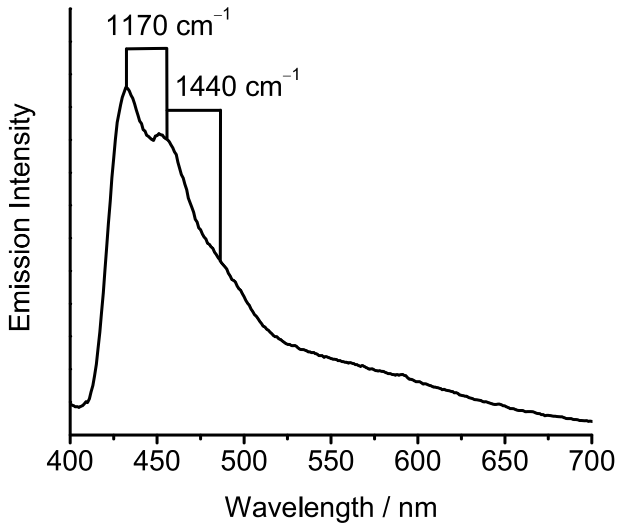

Figure S22: Emission spectrum of **1 (CNC)Cu^I** in 2-methyltetrahydrofuran glass at 77 K

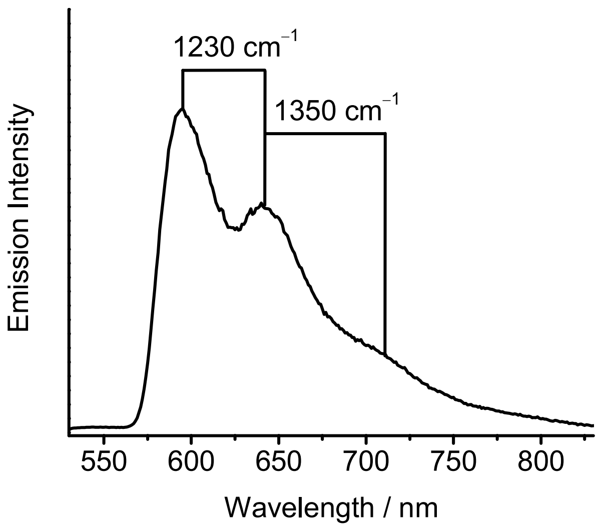

Figure S23: Emission spectrum of **2 (CNC)Ag^{I}** in 2-methyltetrahydrofuran glass at 77 K

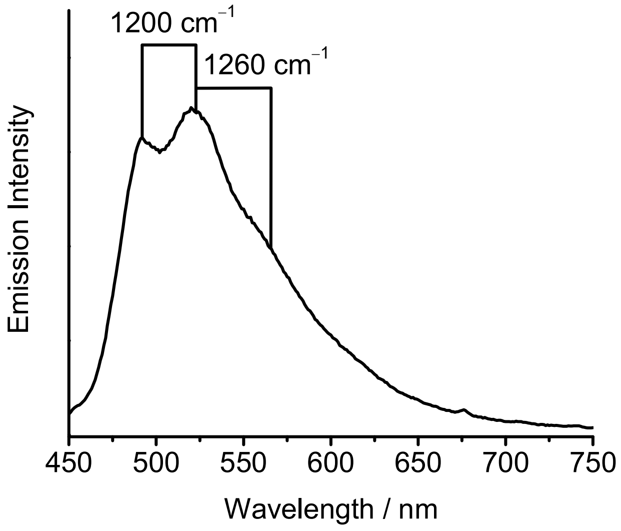

Figure S24: Emission spectrum of **3 (CNC)Au^{I}** in 2-methyltetrahydrofuran glass at 77 K

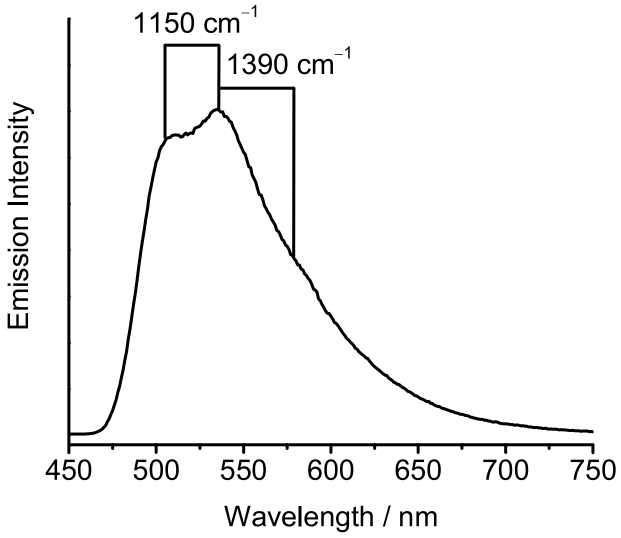

Figure S25: Emission spectrum of **4 [(CN^{Me}C)Au^{I}]OTf** in 2-methyltetrahydrofuran glass at 77 K

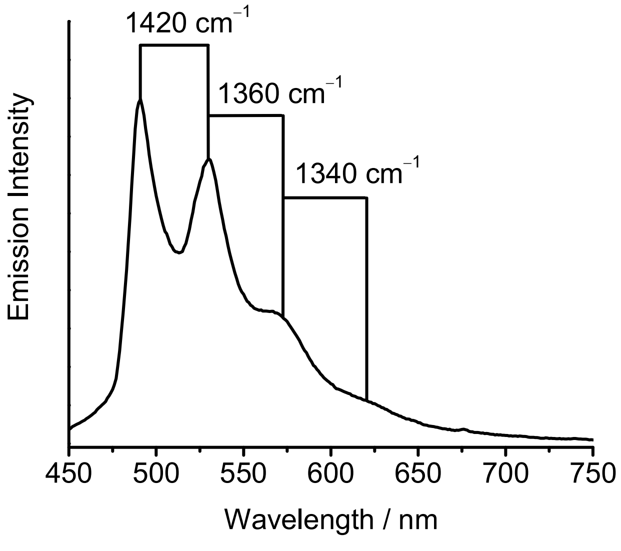

Figure S26: Emission spectrum of **5 [(CNC)Au^{III}]BF$_4$** in 2-methyltetrahydrofuran glass at 77 K

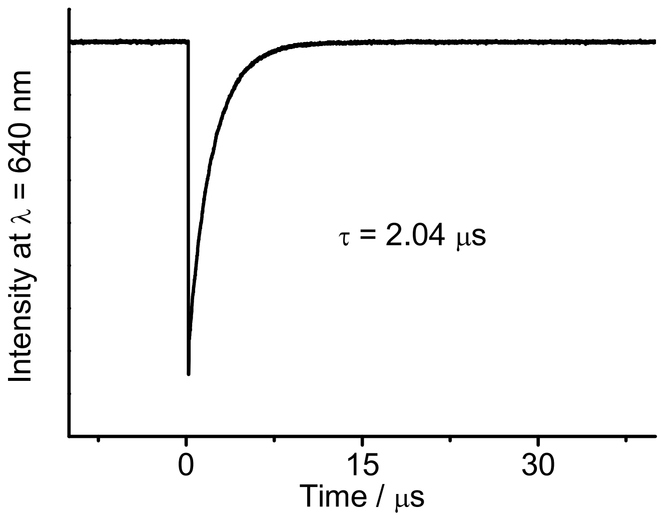

Figure S27: Photoluminescence decay profile of **2 (CNC)Ag^{I}** in degassed THF at 298 K

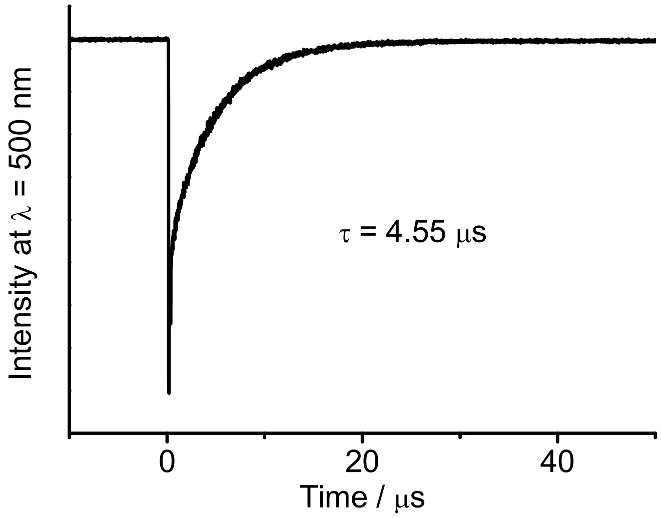

Figure S28: Photoluminescence decay profile of **3 (CNC)Au^{I}** in degassed THF at 298 K

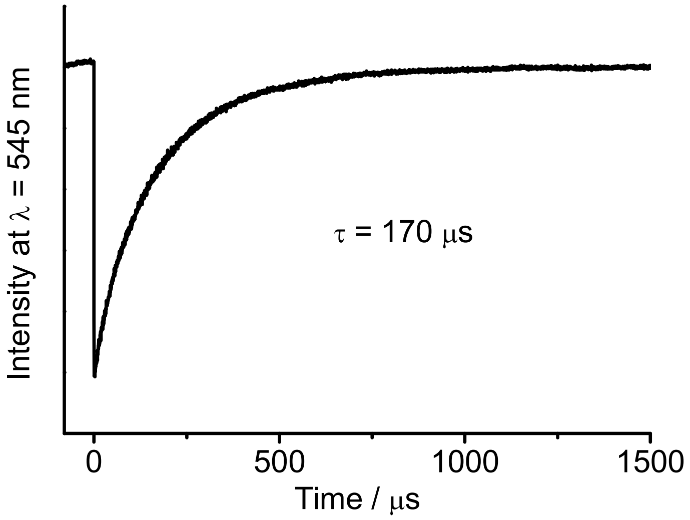

Figure S29: Photoluminescence decay profile of **4 [(CN^{Me}C)Au^{I}]OTf** in degassed THF at 298 K

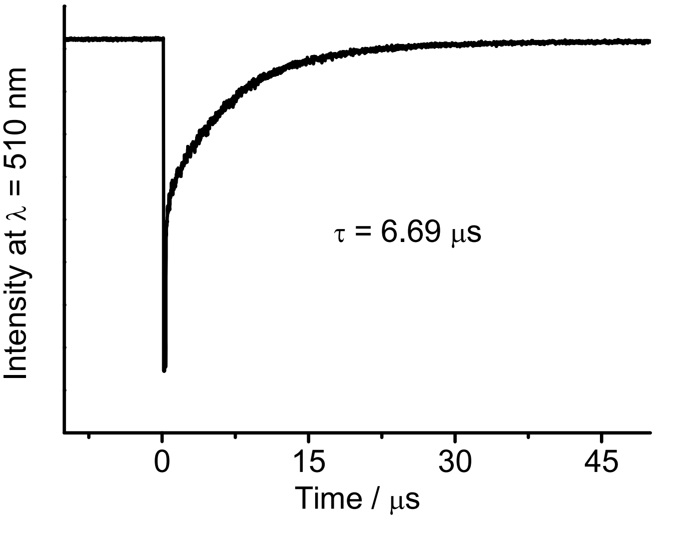

Figure S30: Photoluminescence decay profile of **5 [(CNC)Au^{III}]BF$_4$** in degassed THF at 298 K

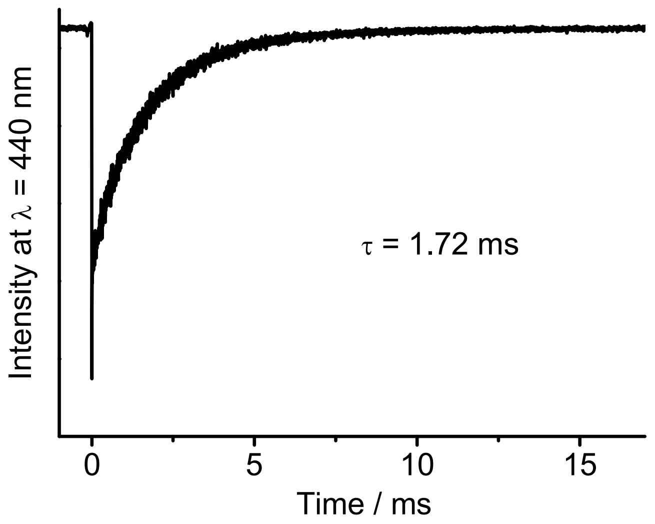

Figure S31: Photoluminescence decay profile of **1 (CNC)Cu^{I}** in butyronitrile glass at 77 K

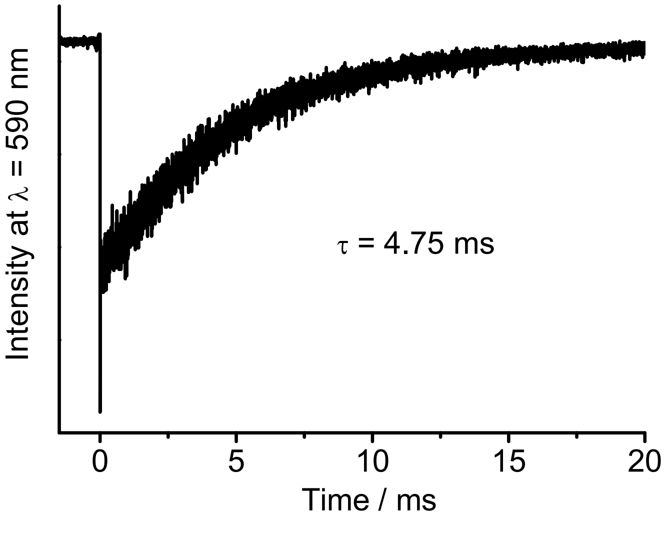

Figure S32: Photoluminescence decay profile of **2 (CNC)Ag^{I}** in butyronitrile glass at 77 K

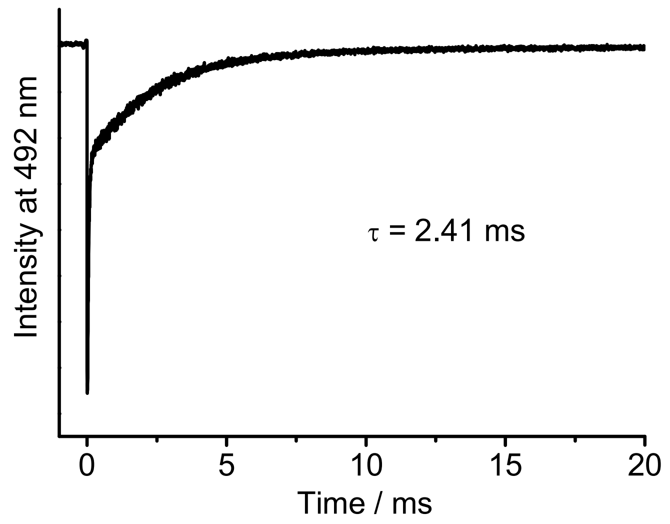

Figure S33: Photoluminescence decay profile of **3 (CNC)Au^{I}** in butyronitrile glass at 77 K

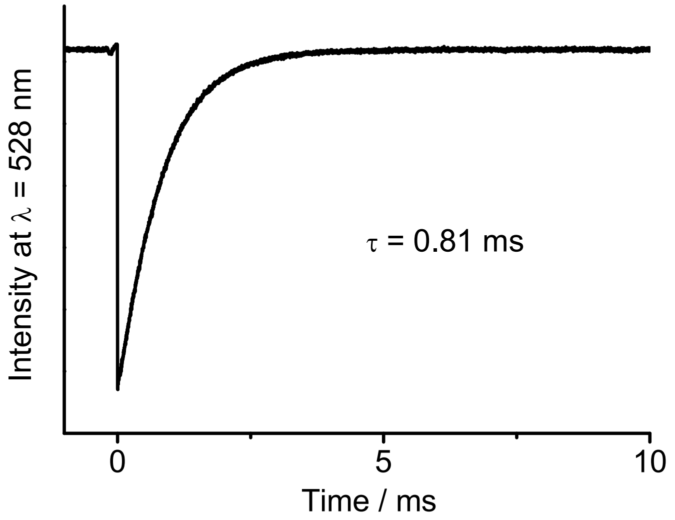

Figure S34: Photoluminescence decay profile of **4 [(CN^{Me}C)Au^{I}]OTf** in butyronitrile glass at 77 K

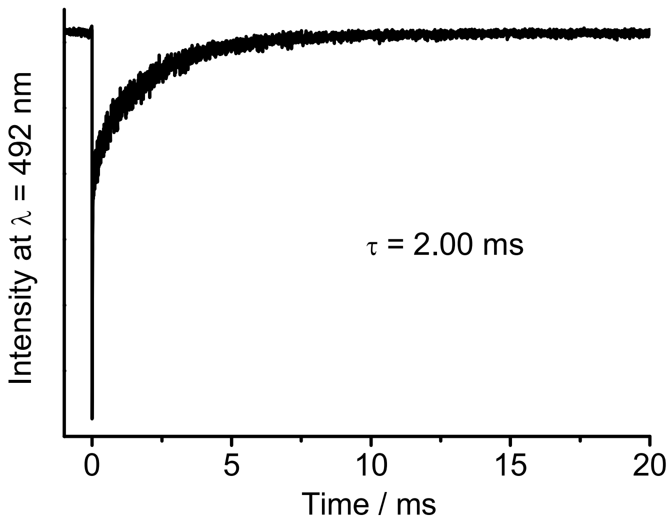

Figure S35: Photoluminescence decay profile of **5 [(CNC)Au^{III}]BF$_4$** in butyronitrile glass at 77 K

Section S6: Computational Details

All the calculations reported in this paper were obtained with the GAUSSIAN 09 suite of programs.[11] Electron correlation was partially taken into account using the B3LYP[12] functional (uB3LYP for triplet species) in conjuction with the D3 dispersion correction suggested by Grimme et al.[13] and the double-ζ quality plus polarization functions def2-SVP[14] basis set for all atoms. All species were characterized by frequency calculations,[15] and have positive definite Hessian matrices. In all species, the bulky *t*-butyl groups were replaced by methyl groups.

Calculations of the absorption spectrum were accomplished using time-dependent density functional theory (TD-DFT)[16] at the same DFT level using the optimized geometries. The assignment of the excitation energies to the experimental bands was performed on the basis of the energy values and oscillator strengths. The B3LYP Hamiltonian was chosen because it was proven to provide reasonable UV-vis spectra for a variety of chromophores including transition metal complexes.[17] For the TD-DFT calculations, solvent effects (solvent = tetrahydrofuran) were taken into account by using the Polarizable Continuum Model (PCM).[18] This level is denoted PCM(THF)-B3LYP-D3/def2-SVP// B3LYP-D3/def2-SVP. Cartesian coordinates (in Å) and total energies (in a. u., ZPVE included) of all the stationary points discussed in the text. All calculations have been performed at the B3LYP-D3/def2-SVP level.

Table S1: Comparison of experimental vs calculated electronic absorptions

Complex	Electronic Absorption		
	**λ_{max}/nm (exp) (ε /dm^3 mol^{-1}cm^{-1})**	**λ_{max}/nm (calculated) (*f, oscillator strength*)**	**Transition**
**1**	506 (7520)	497 (0.074)	HOMO–1→LUMO
	464 (2310)	457 (0.122)	HOMO–1→LUMO+1
**2**	451 (8820)	472 (0.159)	HOMO–1→LUMO+1
	506 (7520)	498 (0.074)	HOMO–1→LUMO
**3**	443 (8360)	479 (0.142)	HOMO–1→LUMO+1
	498 (8150)	504 (0.064)	HOMO–1→LUMO
**4**	366 (6280)	395 (0.074)	HOMO→LUMO+1
	399 (5530)	402 (0.044)	HOMO→LUMO
**5**	372 (6480)	391 (0.135)	HOMO–1→LUMO+2
	405 (5340)	407 (0.057)	HOMO→LUMO+1

Table S2: TD-DFT Calculated energies (eV) of the transitions

**Complex**	**$\Delta E(T_1–S_0)$**	**$\Delta E(S_1–S_0)$**	**$\Delta E(T_1–S_1)$**
**1**	1.851	2.024	0.173
**2**	2.041	2.248	0.207
**3**	2.027	2.152	0.125
**4**	2.663	3.082	0.419
**5**	2.447	2.682	0.235

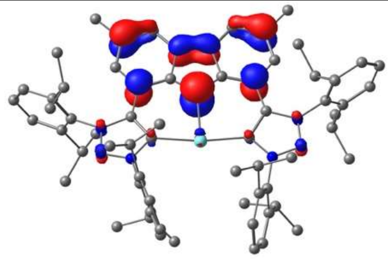	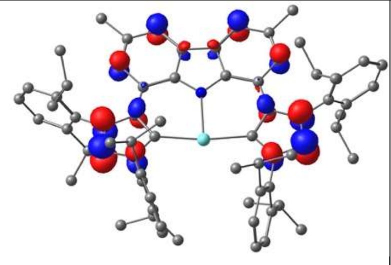	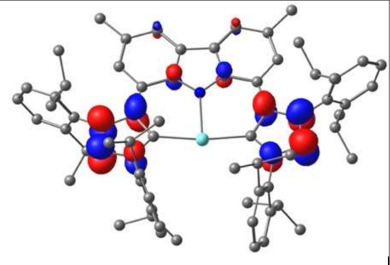
HOMO–1 (**2**)	LUMO (**2**)	LUMO+1 (**2**)
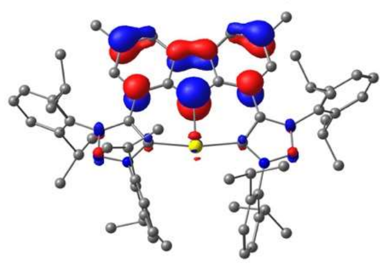	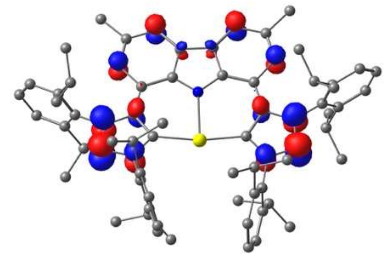	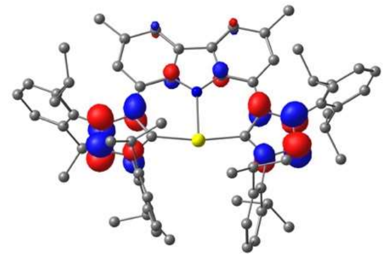
HOMO–1 (**3**)	LUMO (**3**)	LUMO+1 (**3**)
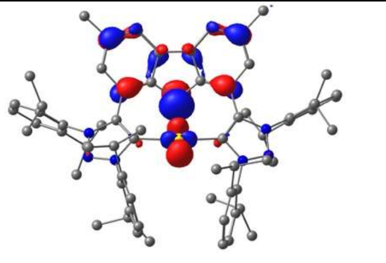	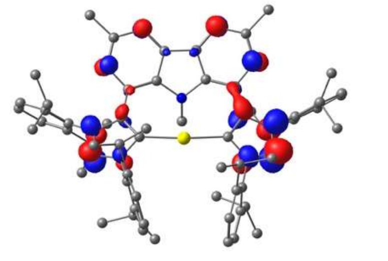	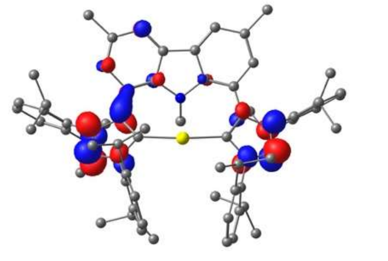
HOMO (**4**)	LUMO (**4**)	LUMO+1 (**4**)
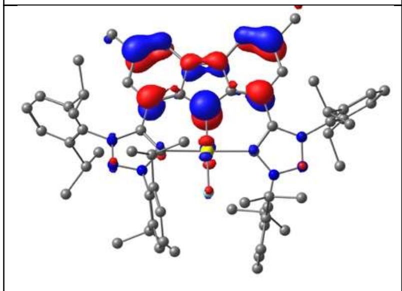	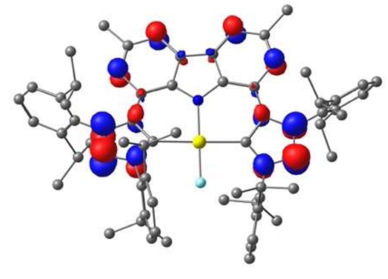	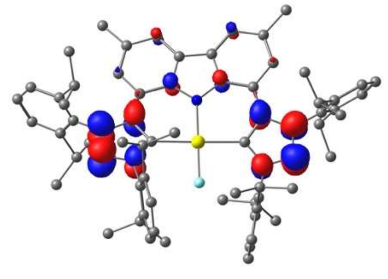
HOMO (**5**)	LUMO+1 (**5**)	LUMO+2 (**5**)

Figure S36: Calculated MOs involved in the lowest energy absorptions

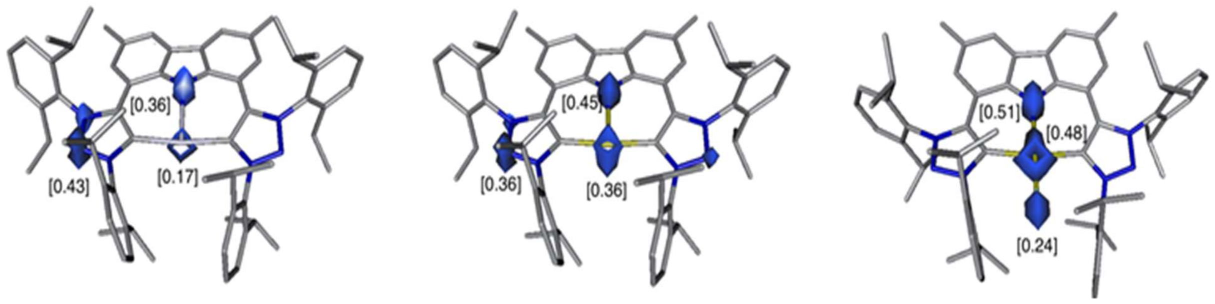

2 (T_1) **3** (T_1) **5** (T_1)

Figure S37: Computed spin densities of the optimized structures of the triplet states of **2**, **3** and **5**

Table S3: Computed (uB3LYP/def2-SVP) T_1–S_0 energy differences (eV)

**Complex**	**ΔE(T_1–S_0)**
**1**	1.611
**2**	1.710
**3**	1.321
**4**	2.352
**5**	1.652

1: E= -4582.446824

Cu	0.000002000	0.236889000	-0.000025000
N	0.000011000	-1.833610000	-0.000025000
N	2.814624000	1.326446000	0.208650000
N	4.098232000	1.003909000	0.214367000
N	4.088164000	-0.303239000	0.016435000
N	-2.814633000	1.326427000	-0.208671000
N	-4.098239000	1.003877000	-0.214371000
N	-4.088156000	-0.303271000	-0.016436000
C	1.089661000	-2.636968000	-0.086642000
C	2.458763000	-2.245443000	-0.180019000
C	3.396939000	-3.295646000	-0.292413000
H	4.454157000	-3.064879000	-0.390038000
C	3.045226000	-4.657480000	-0.287908000
C	1.693853000	-5.021128000	-0.164217000
H	1.412215000	-6.079109000	-0.153244000
C	0.721535000	-4.028836000	-0.063355000
C	-0.721494000	-4.028842000	0.063296000
C	-1.693805000	-5.021142000	0.164154000
H	-1.412159000	-6.079120000	0.153176000
C	-3.045181000	-4.657504000	0.287850000
C	-3.396903000	-3.295674000	0.292364000
H	-4.454123000	-3.064916000	0.389997000
C	-2.458736000	-2.245463000	0.179971000
C	-1.089631000	-2.636977000	0.086588000
C	2.807710000	-0.823403000	-0.096536000
C	1.937869000	0.295915000	0.015008000
C	-2.807695000	-0.823425000	0.096502000
C	-1.937866000	0.295902000	-0.015049000
C	2.447956000	2.683852000	0.520951000
C	2.236182000	3.005017000	1.874010000
C	1.872546000	4.327447000	2.166143000
H	1.699203000	4.621141000	3.203027000
C	1.719441000	5.269023000	1.149563000
H	1.424378000	6.291962000	1.397336000
C	1.933542000	4.914549000	-0.183238000
H	1.799633000	5.664787000	-0.963024000
C	2.310561000	3.610528000	-0.529456000
C	2.336567000	1.954256000	2.974278000
H	2.884256000	1.092119000	2.567939000
C	3.126858000	2.446149000	4.194979000
H	3.248402000	1.626990000	4.921498000
H	2.609547000	3.267478000	4.716729000
H	4.129340000	2.801036000	3.909929000
C	0.936517000	1.454170000	3.372720000
H	0.382623000	1.097391000	2.490175000
H	0.351208000	2.259429000	3.843807000

H	1.013586000	0.621840000	4.090885000
C	2.593041000	3.202222000	-1.970074000
H	2.262627000	2.155170000	-2.072601000
C	4.104611000	3.258921000	-2.267399000
H	4.680709000	2.646244000	-1.560306000
H	4.469810000	4.296659000	-2.193961000
H	4.311706000	2.896437000	-3.287379000
C	1.811400000	4.026705000	-2.998895000
H	1.924697000	3.584155000	-4.000999000
H	2.182173000	5.063134000	-3.058586000
H	0.741040000	4.059407000	-2.754285000
C	5.374387000	-0.943214000	-0.068386000
C	5.933917000	-1.465664000	1.112519000
C	7.176826000	-2.100590000	1.000851000
H	7.649968000	-2.521753000	1.889402000
C	7.816890000	-2.208847000	-0.235972000
H	8.783122000	-2.715382000	-0.303132000
C	7.233006000	-1.677151000	-1.385764000
H	7.745530000	-1.773853000	-2.345149000
C	5.993542000	-1.023396000	-1.327923000
C	5.217445000	-1.323409000	2.448407000
H	4.144218000	-1.201107000	2.235792000
C	5.348059000	-2.566544000	3.337767000
H	4.713673000	-2.457114000	4.231389000
H	6.381431000	-2.717884000	3.689778000
H	5.028649000	-3.473535000	2.801385000
C	5.693144000	-0.050207000	3.171704000
H	5.142777000	0.089548000	4.115594000
H	5.534159000	0.840275000	2.545442000
H	6.768106000	-0.115382000	3.407796000
C	5.328387000	-0.485225000	-2.588818000
H	4.483070000	0.147591000	-2.282392000
C	4.755290000	-1.638662000	-3.431691000
H	4.033593000	-2.235789000	-2.854357000
H	5.558793000	-2.312819000	-3.771770000
H	4.240627000	-1.245783000	-4.323448000
C	6.272956000	0.403474000	-3.411332000
H	7.120452000	-0.168524000	-3.822077000
H	6.678837000	1.226059000	-2.802790000
H	5.731639000	0.844415000	-4.263450000
C	-2.447985000	2.683840000	-0.520965000
C	-2.310621000	3.610516000	0.529445000
C	-1.933630000	4.914548000	0.183236000
H	-1.799746000	5.664787000	0.963025000
C	-1.719531000	5.269033000	-1.149563000
H	-1.424491000	6.291980000	-1.397329000
C	-1.872609000	4.327459000	-2.166147000
H	-1.699270000	4.621161000	-3.203029000
C	-2.236212000	3.005017000	-1.874023000

C	-2.593108000	3.202204000	1.970060000
H	-2.262692000	2.155153000	2.072587000
C	-1.811476000	4.026689000	2.998886000
H	-1.924772000	3.584135000	4.000988000
H	-2.182258000	5.063116000	3.058580000
H	-0.741115000	4.059401000	2.754279000
C	-4.104681000	3.258902000	2.267374000
H	-4.680774000	2.646235000	1.560268000
H	-4.469877000	4.296641000	2.193946000
H	-4.311784000	2.896406000	3.287348000
C	-2.336571000	1.954265000	-2.974301000
H	-2.884212000	1.092097000	-2.567962000
C	-0.936505000	1.454252000	-3.372780000
H	-1.013550000	0.621933000	-4.090961000
H	-0.382579000	1.097479000	-2.490253000
H	-0.351242000	2.259547000	-3.843861000
C	-3.126910000	2.446140000	-4.194978000
H	-3.248430000	1.626987000	-4.921508000
H	-2.609648000	3.267502000	-4.716724000
H	-4.129403000	2.800975000	-3.909901000
C	-5.374373000	-0.943251000	0.068427000
C	-5.993496000	-1.023413000	1.327983000
C	-7.232958000	-1.677169000	1.385864000
H	-7.745457000	-1.773859000	2.345263000
C	-7.816870000	-2.208883000	0.236095000
H	-8.783099000	-2.715418000	0.303288000
C	-7.176839000	-2.100641000	-1.000747000
H	-7.650004000	-2.521817000	-1.889279000
C	-5.933934000	-1.465716000	-1.112456000
C	-5.328297000	-0.485231000	2.588850000
H	-4.483042000	0.147649000	2.282384000
C	-4.755069000	-1.638655000	3.431653000
H	-5.558507000	-2.312882000	3.771746000
H	-4.240390000	-1.245769000	4.323398000
H	-4.033358000	-2.235709000	2.854262000
C	-6.272862000	0.403386000	3.411456000
H	-6.678871000	1.225940000	2.802956000
H	-5.731501000	0.844369000	4.263524000
H	-7.120270000	-0.168683000	3.822284000
C	-5.217495000	-1.323475000	-2.448362000
H	-4.144259000	-1.201200000	-2.235775000
C	-5.348162000	-2.566603000	-3.337724000
H	-6.381548000	-2.717915000	-3.689709000
H	-5.028762000	-3.473605000	-2.801354000
H	-4.713798000	-2.457185000	-4.231362000
C	-5.693181000	-0.050259000	-3.171642000
H	-6.768151000	-0.115406000	-3.407705000
H	-5.142836000	0.089485000	-4.115547000
H	-5.534157000	0.840219000	-2.545382000

C	4.124292000	-5.713823000	-0.371129000
H	4.437311000	-6.054870000	0.632596000
H	3.778248000	-6.604652000	-0.919558000
H	5.025413000	-5.334947000	-0.878860000
C	-4.124241000	-5.713853000	0.371065000
H	-4.437312000	-6.054842000	-0.632663000
H	-3.778169000	-6.604713000	0.919426000
H	-5.025337000	-5.335007000	0.878864000

1(T1): E= -4582.387614

Cu	0.000290000	0.114615000	0.000862000
N	0.000628000	-1.817308000	0.008110000
N	2.738979000	1.304678000	0.303739000
N	4.053191000	1.024775000	0.372003000
N	4.093886000	-0.304799000	0.132505000
N	-2.738370000	1.304006000	-0.304631000
N	-4.052584000	1.024293000	-0.371545000
N	-4.093419000	-0.304961000	-0.130874000
C	1.107374000	-2.627150000	-0.095317000
C	2.477079000	-2.256211000	-0.176299000
C	3.407113000	-3.309628000	-0.312444000
H	4.465504000	-3.079263000	-0.411855000
C	3.032616000	-4.667382000	-0.322130000
C	1.682838000	-5.011621000	-0.185756000
H	1.381418000	-6.062852000	-0.168360000
C	0.718637000	-3.999860000	-0.076396000
C	-0.718578000	-4.000102000	0.072930000
C	-1.683002000	-5.012200000	0.177312000
H	-1.381693000	-6.063387000	0.155841000
C	-3.032837000	-4.668357000	0.313837000
C	-3.407181000	-3.310516000	0.308483000
H	-4.465819000	-3.080477000	0.405767000
C	-2.476831000	-2.256704000	0.177973000
C	-1.106921000	-2.627413000	0.099960000
C	2.822736000	-0.851087000	-0.040617000
C	1.935647000	0.222129000	0.061083000
C	-2.822350000	-0.851509000	0.041479000
C	-1.935006000	0.221234000	-0.063081000
C	2.302938000	2.630309000	0.623478000
C	1.775442000	2.862881000	1.910388000
C	1.352130000	4.166611000	2.206279000
H	0.948317000	4.387277000	3.196596000
C	1.452822000	5.185878000	1.261681000
H	1.109026000	6.193088000	1.510219000
C	1.990105000	4.928321000	-0.000758000
H	2.059500000	5.739012000	-0.727494000
C	2.432899000	3.645873000	-0.347858000
C	1.678459000	1.763584000	2.962749000

H	2.131341000	0.853514000	2.546412000
C	2.472674000	2.122284000	4.228819000
H	2.448676000	1.284850000	4.944674000
H	2.052055000	3.005557000	4.736197000
H	3.525195000	2.336043000	3.989325000
C	0.214752000	1.428248000	3.296240000
H	-0.363222000	1.189455000	2.388445000
H	-0.288134000	2.270904000	3.796362000
H	0.159700000	0.555484000	3.965900000
C	3.067664000	3.358989000	-1.703265000
H	2.891164000	2.292798000	-1.921936000
C	4.591820000	3.580265000	-1.646920000
H	5.050920000	2.965114000	-0.861480000
H	4.817463000	4.639336000	-1.438725000
H	5.055935000	3.315550000	-2.611040000
C	2.445918000	4.170775000	-2.847010000
H	2.811662000	3.798677000	-3.817041000
H	2.718052000	5.237057000	-2.786603000
H	1.349910000	4.100363000	-2.838893000
C	5.389514000	-0.913238000	0.102401000
C	5.842536000	-1.586021000	1.255583000
C	7.104571000	-2.190660000	1.189818000
H	7.492438000	-2.730456000	2.054819000
C	7.875255000	-2.113953000	0.028067000
H	8.856277000	-2.595201000	-0.003349000
C	7.404976000	-1.428743000	-1.093209000
H	8.022467000	-1.382292000	-1.991700000
C	6.147423000	-0.809175000	-1.080324000
C	4.987772000	-1.624432000	2.516563000
H	3.934524000	-1.628449000	2.197256000
C	5.193655000	-2.889846000	3.356981000
H	4.458419000	-2.920322000	4.176338000
H	6.194275000	-2.923785000	3.818248000
H	5.061829000	-3.798272000	2.748737000
C	5.211041000	-0.348598000	3.350700000
H	4.547037000	-0.338653000	4.230388000
H	5.007541000	0.553394000	2.754729000
H	6.252953000	-0.294812000	3.707451000
C	5.579342000	-0.113441000	-2.311902000
H	4.876898000	0.653969000	-1.956775000
C	4.781600000	-1.111724000	-3.171612000
H	3.981654000	-1.596569000	-2.592917000
H	5.441780000	-1.904437000	-3.561028000
H	4.319438000	-0.599476000	-4.031394000
C	6.641615000	0.614228000	-3.145096000
H	7.334816000	-0.086256000	-3.638684000
H	7.235823000	1.303939000	-2.525794000
H	6.156776000	1.201949000	-3.940870000
C	-2.302336000	2.629319000	-0.625873000

C	-2.431575000	3.645798000	0.344633000
C	-1.988335000	4.927740000	-0.003791000
H	-2.057094000	5.739121000	0.722223000
C	-1.451312000	5.183923000	-1.266614000
H	-1.107033000	6.190729000	-1.516112000
C	-1.351573000	4.163822000	-2.210406000
H	-0.948089000	4.383460000	-3.201084000
C	-1.775479000	2.860569000	-1.913280000
C	-3.065953000	3.360478000	1.700583000
H	-2.888513000	2.294765000	1.920883000
C	-2.444868000	4.174632000	2.843004000
H	-2.809807000	3.803394000	3.813664000
H	-2.718498000	5.240451000	2.781193000
H	-1.348776000	4.105749000	2.834631000
C	-4.590320000	3.580397000	1.644278000
H	-5.049199000	2.963453000	0.860132000
H	-4.816845000	4.638915000	1.434250000
H	-5.053885000	3.317072000	2.609042000
C	-1.680314000	1.760610000	-2.965141000
H	-2.133204000	0.851023000	-2.547746000
C	-0.217262000	1.424190000	-3.300345000
H	-0.163812000	0.552114000	-3.971050000
H	0.361145000	1.183783000	-2.393283000
H	0.285970000	2.266827000	-3.800175000
C	-2.476039000	2.119210000	-4.230323000
H	-2.453693000	1.281343000	-4.945728000
H	-2.055430000	3.001857000	-4.738797000
H	-3.528061000	2.333955000	-3.989500000
C	-5.389525000	-0.912356000	-0.098238000
C	-6.144672000	-0.808284000	1.086201000
C	-7.402988000	-1.426293000	1.101337000
H	-8.018412000	-1.379893000	2.001251000
C	-7.876672000	-2.109780000	-0.019546000
H	-8.858270000	-2.589735000	0.013582000
C	-7.108654000	-2.186446000	-1.183071000
H	-7.499079000	-2.724953000	-2.047732000
C	-5.845915000	-1.583541000	-1.250995000
C	-5.573072000	-0.114208000	2.317058000
H	-4.869240000	0.651575000	1.961109000
C	-4.776540000	-1.114729000	3.175276000
H	-5.438199000	-1.905685000	3.565755000
H	-4.311455000	-0.603863000	4.034304000
H	-3.978972000	-1.601690000	2.595052000
C	-6.632374000	0.615904000	3.151902000
H	-7.225644000	1.307295000	2.533580000
H	-6.145019000	1.202174000	3.947211000
H	-7.326685000	-0.082980000	3.646191000
C	-4.993653000	-1.622598000	-2.513645000
H	-3.939777000	-1.626139000	-2.196401000

C	-5.200989000	-2.888822000	-3.352503000
H	-6.202557000	-2.923435000	-3.811652000
H	-5.067701000	-3.796612000	-2.743615000
H	-4.467438000	-2.919945000	-4.173345000
C	-5.218777000	-0.347441000	-3.348289000
H	-6.261492000	-0.293852000	-3.702716000
H	-4.556759000	-0.338231000	-4.229482000
H	-5.013805000	0.554962000	-2.753440000
C	4.100073000	-5.724804000	-0.488597000
H	4.964288000	-5.532639000	0.168994000
H	3.715909000	-6.729817000	-0.256585000
H	4.485323000	-5.748130000	-1.523244000
C	-4.100652000	-5.726294000	0.474635000
H	-4.964032000	-5.531328000	-0.183248000
H	-3.716340000	-6.730396000	0.238947000
H	-4.487217000	-5.753853000	1.508687000

2: E= -3089.085682

Ag	0.000087000	-0.506483000	-0.000822000
N	-0.000110000	1.831273000	0.000541000
N	3.023599000	-1.331632000	-0.155750000
N	4.280800000	-0.950206000	-0.016444000
N	4.192514000	0.364687000	0.104957000
N	-3.023339000	-1.331897000	0.155600000
N	-4.280701000	-0.950585000	0.017373000
N	-4.192642000	0.364310000	-0.104155000
C	1.095414000	2.627622000	0.023286000
C	2.475639000	2.247245000	0.050072000
C	3.405975000	3.313353000	0.058516000
H	4.469084000	3.095685000	0.066677000
C	3.049275000	4.671322000	0.053160000
C	1.688940000	5.023715000	0.032028000
H	1.397249000	6.079310000	0.025990000
C	0.723578000	4.023242000	0.014144000
C	-0.723890000	4.023183000	-0.016666000
C	-1.689283000	5.023592000	-0.036162000
H	-1.397632000	6.079205000	-0.031600000
C	-3.049594000	4.671110000	-0.057167000
C	-3.406231000	3.313118000	-0.061052000
H	-4.469330000	3.095435000	-0.069535000
C	-2.475867000	2.247045000	-0.050932000
C	-1.095666000	2.627528000	-0.023798000
C	2.883127000	0.834264000	0.029116000
C	2.093880000	-0.335264000	-0.121167000
C	-2.883238000	0.834032000	-0.029388000
C	-2.093750000	-0.335428000	0.120198000
C	2.729767000	-2.717269000	-0.426099000

C	2.821954000	-3.154131000	-1.760153000
C	2.472185000	-4.487188000	-2.020226000
H	2.519807000	-4.867337000	-3.042197000
C	2.044235000	-5.327463000	-0.993294000
H	1.765290000	-6.360228000	-1.218076000
C	1.958445000	-4.858063000	0.318555000
H	1.608742000	-5.528722000	1.103738000
C	2.302134000	-3.537602000	0.635295000
C	3.195599000	-2.200233000	-2.889435000
H	3.743242000	-1.355523000	-2.448965000
C	4.130715000	-2.833002000	-3.928705000
H	4.452152000	-2.072597000	-4.657947000
H	3.633841000	-3.635556000	-4.497336000
H	5.031105000	-3.255845000	-3.456589000
C	1.925388000	-1.630714000	-3.547650000
H	1.276078000	-1.147606000	-2.801814000
H	1.347450000	-2.431568000	-4.035798000
H	2.189557000	-0.882822000	-4.313052000
C	2.243024000	-3.002723000	2.060498000
H	1.918199000	-1.952379000	1.992149000
C	3.642423000	-3.022327000	2.705387000
H	4.374037000	-2.468505000	2.099345000
H	4.005822000	-4.057916000	2.811965000
H	3.612794000	-2.566235000	3.708282000
C	1.217581000	-3.725217000	2.940554000
H	1.116263000	-3.200813000	3.903535000
H	1.524378000	-4.759991000	3.165911000
H	0.227667000	-3.756116000	2.462177000
C	5.431421000	1.045411000	0.378709000
C	6.228646000	1.442243000	-0.710990000
C	7.424755000	2.107926000	-0.416603000
H	8.075893000	2.434180000	-1.229568000
C	7.792186000	2.367605000	0.905863000
H	8.725284000	2.897049000	1.114827000
C	6.978699000	1.955573000	1.961076000
H	7.280055000	2.166835000	2.989244000
C	5.776748000	1.273799000	1.722015000
C	5.803906000	1.147192000	-2.142997000
H	4.717205000	0.969556000	-2.133844000
C	6.049565000	2.326286000	-3.094068000
H	5.623386000	2.105959000	-4.085573000
H	7.124031000	2.524122000	-3.238485000
H	5.577476000	3.246502000	-2.716323000
C	6.478521000	-0.142485000	-2.644540000
H	6.134625000	-0.391363000	-3.661200000
H	6.245437000	-0.991017000	-1.984042000
H	7.574119000	-0.022520000	-2.673518000
C	4.879187000	0.843525000	2.875230000
H	4.076596000	0.213283000	2.465519000

C	4.211784000	2.063727000	3.534230000
H	3.638317000	2.649557000	2.800463000
H	4.966348000	2.727826000	3.987524000
H	3.522137000	1.741050000	4.330810000
C	5.636441000	-0.013313000	3.901697000
H	6.425324000	0.562242000	4.412650000
H	6.107081000	-0.886762000	3.424698000
H	4.942980000	-0.380197000	4.675214000
C	-2.729194000	-2.717493000	0.425877000
C	-2.301492000	-3.537717000	-0.635578000
C	-1.957614000	-4.858151000	-0.318929000
H	-1.607850000	-5.528716000	-1.104164000
C	-2.043348000	-5.327661000	0.992881000
H	-1.764309000	-6.360420000	1.217579000
C	-2.471380000	-4.487507000	2.019877000
H	-2.518966000	-4.867747000	3.041813000
C	-2.821271000	-3.154461000	1.759916000
C	-2.242589000	-3.002786000	-2.060766000
H	-1.917684000	-1.952474000	-1.992428000
C	-1.217337000	-3.725289000	-2.941038000
H	-1.116148000	-3.200827000	-3.904002000
H	-1.524211000	-4.760040000	-3.166403000
H	-0.227339000	-3.756245000	-2.462831000
C	-3.642116000	-3.022265000	-2.705364000
H	-4.373569000	-2.468446000	-2.099119000
H	-4.005619000	-4.057818000	-2.811934000
H	-3.612683000	-2.566103000	-3.708233000
C	-3.194889000	-2.200646000	2.889280000
H	-3.743085000	-1.356224000	2.448959000
C	-1.924669000	-1.630525000	3.546972000
H	-2.188882000	-0.882553000	4.312278000
H	-1.275724000	-1.147358000	2.800860000
H	-1.346332000	-2.431073000	4.035139000
C	-4.129296000	-2.833699000	3.928999000
H	-4.450806000	-2.073326000	4.658237000
H	-3.631828000	-3.635918000	4.497589000
H	-5.029675000	-3.257054000	3.457316000
C	-5.431836000	1.044901000	-0.376934000
C	-5.778641000	1.272481000	-1.720009000
C	-6.980731000	1.954314000	-1.958165000
H	-7.283205000	2.164961000	-2.986133000
C	-7.792954000	2.367189000	-0.902298000
H	-8.726179000	2.896687000	-1.110558000
C	-7.424087000	2.108281000	0.419914000
H	-8.074245000	2.435165000	1.233410000
C	-6.227775000	1.442551000	0.713387000
C	-4.882443000	0.841348000	-2.873964000
H	-4.079438000	0.211306000	-2.464770000
C	-4.215660000	2.061022000	-3.534571000

H	-4.970671000	2.725012000	-3.987272000
H	-3.527122000	1.737731000	-4.331861000
H	-3.641103000	2.647187000	-2.801918000
C	-5.641032000	-0.016066000	-3.898958000
H	-6.111305000	-0.889082000	-3.420804000
H	-4.948539000	-0.383630000	-4.673017000
H	-6.430390000	0.559302000	-4.409390000
C	-5.801596000	1.148219000	2.145106000
H	-4.714992000	0.970066000	2.134899000
C	-6.045699000	2.328044000	3.095670000
H	-7.119921000	2.526528000	3.241022000
H	-5.573514000	3.247763000	2.716837000
H	-5.618636000	2.108143000	4.086891000
C	-6.476313000	-0.140821000	2.648128000
H	-7.571816000	-0.020274000	2.678286000
H	-6.131393000	-0.389319000	3.664534000
H	-6.244448000	-0.989843000	1.987824000
C	4.116962000	5.741902000	0.068070000
H	4.044638000	6.400855000	-0.814579000
H	4.028700000	6.390527000	0.956970000
H	5.127974000	5.305725000	0.076103000
C	-4.117330000	5.741624000	-0.073306000
H	-4.047334000	6.399334000	0.810480000
H	-4.026825000	6.391515000	-0.961033000
H	-5.128281000	5.305376000	-0.084465000

2(T1): E= -3089.022776

Ag	0.002948000	0.450349000	-0.327788000
N	0.032717000	-1.845245000	-0.054158000
N	-2.995537000	1.316596000	-0.118003000
N	-4.234046000	0.932521000	0.152157000
N	-4.147861000	-0.387976000	0.200389000
N	2.965970000	1.388821000	-0.045694000
N	4.281273000	1.013700000	0.030221000
N	4.214032000	-0.337399000	-0.166340000
C	-1.085146000	-2.618516000	-0.254262000
C	-2.456624000	-2.255033000	-0.168337000
C	-3.392452000	-3.304372000	-0.337011000
H	-4.454911000	-3.087264000	-0.285270000
C	-3.022642000	-4.631799000	-0.604251000
C	-1.661991000	-4.956205000	-0.755924000
H	-1.364873000	-5.980422000	-0.999382000
C	-0.704755000	-3.956880000	-0.608137000
C	0.739349000	-3.925914000	-0.749304000
C	1.688651000	-4.891207000	-1.080477000
H	1.399229000	-5.929298000	-1.264561000
C	3.043005000	-4.497504000	-1.177795000
C	3.408750000	-3.169700000	-0.934920000

H	4.456245000	-2.907888000	-1.056486000
C	2.482389000	-2.150766000	-0.562175000
C	1.120554000	-2.580487000	-0.449964000
C	-2.862100000	-0.850495000	-0.047642000
C	-2.088303000	0.307865000	-0.255726000
C	2.880105000	-0.780119000	-0.349166000
C	2.090554000	0.374689000	-0.298830000
C	-2.711729000	2.716384000	-0.313037000
C	-2.865694000	3.239579000	-1.608697000
C	-2.511718000	4.583321000	-1.799015000
H	-2.601477000	5.028358000	-2.791858000
C	-2.026993000	5.350395000	-0.741168000
H	-1.740857000	6.391173000	-0.912402000
C	-1.891154000	4.798717000	0.534100000
H	-1.495466000	5.414856000	1.341205000
C	-2.233550000	3.463600000	0.781619000
C	-3.328140000	2.374810000	-2.775601000
H	-3.773618000	1.458673000	-2.361798000
C	-4.418723000	3.053451000	-3.616696000
H	-4.787297000	2.360432000	-4.389934000
H	-4.039559000	3.948056000	-4.136094000
H	-5.274449000	3.357780000	-2.994428000
C	-2.126901000	1.952004000	-3.639905000
H	-1.364416000	1.439323000	-3.034065000
H	-1.652100000	2.828677000	-4.108599000
H	-2.448286000	1.267812000	-4.441906000
C	-2.121898000	2.839596000	2.168070000
H	-1.794768000	1.795611000	2.025734000
C	-3.496288000	2.812286000	2.866159000
H	-4.252105000	2.291828000	2.261305000
H	-3.854616000	3.839239000	3.046024000
H	-3.423579000	2.301427000	3.839880000
C	-1.076752000	3.518516000	3.059291000
H	-0.942103000	2.938713000	3.985516000
H	-1.391627000	4.532181000	3.356532000
H	-0.102770000	3.596792000	2.556511000
C	-5.349280000	-1.090194000	0.570420000
C	-6.280989000	-1.387711000	-0.442803000
C	-7.438758000	-2.075267000	-0.059706000
H	-8.190369000	-2.328367000	-0.809402000
C	-7.641004000	-2.449642000	1.271093000
H	-8.547434000	-2.993757000	1.548367000
C	-6.696960000	-2.135416000	2.247875000
H	-6.870562000	-2.437152000	3.282801000
C	-5.525594000	-1.435900000	1.920896000
C	-6.030666000	-0.972543000	-1.886997000
H	-4.945349000	-0.822999000	-2.002216000
C	-6.435669000	-2.052747000	-2.899084000
H	-6.122931000	-1.755908000	-3.912497000

H	-7.526586000	-2.204488000	-2.927323000
H	-5.960155000	-3.018066000	-2.665082000
C	-6.714378000	0.373729000	-2.187234000
H	-6.488357000	0.702705000	-3.214126000
H	-6.369787000	1.153383000	-1.492170000
H	-7.808834000	0.284747000	-2.089138000
C	-4.487309000	-1.104591000	2.985482000
H	-3.756910000	-0.410392000	2.544489000
C	-3.718945000	-2.368695000	3.410496000
H	-3.236841000	-2.851941000	2.547719000
H	-4.397282000	-3.102280000	3.876701000
H	-2.936578000	-2.115317000	4.143602000
C	-5.108230000	-0.384746000	4.192446000
H	-5.809026000	-1.034199000	4.741370000
H	-5.652837000	0.519893000	3.881633000
H	-4.318948000	-0.082553000	4.899072000
C	2.615439000	2.738298000	0.273462000
C	2.131023000	3.581093000	-0.752296000
C	1.745437000	4.879995000	-0.393818000
H	1.351593000	5.556747000	-1.152311000
C	1.867886000	5.329776000	0.920762000
H	1.567935000	6.349637000	1.176463000
C	2.370927000	4.485207000	1.910953000
H	2.456254000	4.854047000	2.933850000
C	2.744597000	3.167125000	1.614692000
C	2.090892000	3.100900000	-2.199389000
H	1.721276000	2.064510000	-2.186052000
C	1.134720000	3.901862000	-3.089291000
H	1.047328000	3.418461000	-4.075085000
H	1.494542000	4.929744000	-3.262625000
H	0.128093000	3.961021000	-2.648562000
C	3.512322000	3.071494000	-2.794330000
H	4.188389000	2.447934000	-2.191669000
H	3.936871000	4.088465000	-2.836949000
H	3.493073000	2.666302000	-3.819626000
C	3.211900000	2.204293000	2.703181000
H	3.981805000	1.565330000	2.246709000
C	2.057226000	1.292194000	3.160640000
H	2.419931000	0.546329000	3.885515000
H	1.606042000	0.749790000	2.317221000
H	1.265379000	1.881942000	3.647902000
C	3.860051000	2.897181000	3.907416000
H	4.300845000	2.142739000	4.577711000
H	3.128013000	3.469515000	4.501132000
H	4.660857000	3.586461000	3.596974000
C	5.404655000	-1.074959000	0.107351000
C	6.393695000	-1.141380000	-0.898612000
C	7.549394000	-1.885970000	-0.631111000
H	8.333969000	-1.957262000	-1.387139000

C	7.707221000	-2.550194000	0.587412000
H	8.609927000	-3.138119000	0.773774000
C	6.720373000	-2.462536000	1.567987000
H	6.858819000	-2.981753000	2.519521000
C	5.555430000	-1.710110000	1.356635000
C	6.204986000	-0.413227000	-2.222617000
H	5.126672000	-0.221139000	-2.332223000
C	6.645868000	-1.245258000	-3.434667000
H	7.736292000	-1.405920000	-3.453135000
H	6.379512000	-0.725006000	-4.368732000
H	6.156774000	-2.232413000	-3.442739000
C	6.909287000	0.954811000	-2.186347000
H	6.535264000	1.556977000	-1.345960000
H	6.726434000	1.511033000	-3.120586000
H	7.999117000	0.829847000	-2.069657000
C	4.506830000	-1.594333000	2.455703000
H	3.708947000	-0.932306000	2.093566000
C	3.860668000	-2.952975000	2.774128000
H	4.600750000	-3.662333000	3.180573000
H	3.417570000	-3.401781000	1.873021000
H	3.063213000	-2.832666000	3.525424000
C	5.099258000	-0.941313000	3.715813000
H	5.897637000	-1.560493000	4.156099000
H	4.320864000	-0.809225000	4.484764000
H	5.526479000	0.047363000	3.487382000
C	-4.079098000	-5.702377000	-0.736868000
H	-3.903425000	-6.333561000	-1.623349000
H	-4.076159000	-6.373721000	0.139808000
H	-5.088797000	-5.271760000	-0.818551000
C	4.090800000	-5.516174000	-1.554050000
H	4.123691000	-6.338451000	-0.818651000
H	3.868128000	-5.974525000	-2.532922000
H	5.095040000	-5.070168000	-1.605512000

3: E= -3077.873715

Au	0.000015000	-0.499408000	-0.000296000
N	-0.000074000	1.911205000	0.000592000
N	-2.964147000	-1.301795000	0.159130000
N	-4.223837000	-0.922914000	0.066584000
N	-4.147644000	0.389599000	-0.078434000
N	2.964206000	-1.301705000	-0.159180000
N	4.223855000	-0.922801000	-0.066219000
N	4.147589000	0.389707000	0.078801000
C	-1.092255000	2.698898000	-0.051077000
C	-2.462980000	2.294915000	-0.104668000
C	-3.403320000	3.352916000	-0.151319000
H	-4.464106000	3.129515000	-0.192415000
C	-3.056772000	4.713868000	-0.146406000

C	-1.700766000	5.085071000	-0.085105000
H	-1.425272000	6.144981000	-0.074881000
C	-0.724694000	4.097015000	-0.036086000
C	0.724594000	4.097034000	0.035132000
C	1.700690000	5.085132000	0.083146000
H	1.425197000	6.145032000	0.072060000
C	3.056699000	4.713967000	0.144414000
C	3.403239000	3.353006000	0.150068000
H	4.464045000	3.129624000	0.190760000
C	2.462886000	2.294986000	0.104477000
C	1.092128000	2.698934000	0.051288000
C	-2.842639000	0.873008000	-0.063078000
C	-2.041732000	-0.298244000	0.068980000
C	2.842579000	0.873082000	0.063057000
C	2.041739000	-0.298183000	-0.069275000
C	-2.664065000	-2.683090000	0.449174000
C	-2.708060000	-3.088477000	1.794928000
C	-2.371430000	-4.422084000	2.069064000
H	-2.383669000	-4.779386000	3.100307000
C	-1.999971000	-5.290969000	1.044304000
H	-1.729887000	-6.323695000	1.279809000
C	-1.958006000	-4.849988000	-0.279523000
H	-1.649908000	-5.542244000	-1.063313000
C	-2.292517000	-3.531115000	-0.610550000
C	-3.016668000	-2.107174000	2.920300000
H	-3.540014000	-1.245160000	2.483632000
C	-3.950802000	-2.692614000	3.987954000
H	-4.216033000	-1.916459000	4.723184000
H	-3.474958000	-3.515725000	4.544867000
H	-4.882542000	-3.075081000	3.543143000
C	-1.709409000	-1.581790000	3.541687000
H	-1.050519000	-1.151402000	2.772073000
H	-1.161124000	-2.396195000	4.041241000
H	-1.924434000	-0.803043000	4.291457000
C	-2.278332000	-3.024803000	-2.046683000
H	-1.921479000	-1.983442000	-2.009448000
C	-3.701562000	-3.021964000	-2.637597000
H	-4.399074000	-2.443522000	-2.014859000
H	-4.091529000	-4.050554000	-2.713702000
H	-3.699641000	-2.582890000	-3.648485000
C	-1.308276000	-3.790811000	-2.951714000
H	-1.231764000	-3.287618000	-3.928205000
H	-1.650957000	-4.821046000	-3.144147000
H	-0.302367000	-3.837685000	-2.510421000
C	-5.402542000	1.059130000	-0.304370000
C	-6.135960000	1.494364000	0.814519000
C	-7.349058000	2.147852000	0.565793000
H	-7.952416000	2.502973000	1.403112000
C	-7.793648000	2.358014000	-0.741581000

H	-8.738893000	2.878584000	-0.915206000
C	-7.041699000	1.908042000	-1.826712000
H	-7.402937000	2.081105000	-2.842599000
C	-5.825610000	1.237033000	-1.633028000
C	-5.630044000	1.251934000	2.229858000
H	-4.547275000	1.063084000	2.166239000
C	-5.811037000	2.470871000	3.144657000
H	-5.332441000	2.285274000	4.119161000
H	-6.873947000	2.687395000	3.339474000
H	-5.351029000	3.368948000	2.703984000
C	-6.284348000	-0.010463000	2.818930000
H	-5.887401000	-0.221117000	3.824653000
H	-6.091423000	-0.886492000	2.181773000
H	-7.376102000	0.118727000	2.901720000
C	-4.992043000	0.770470000	-2.820016000
H	-4.171724000	0.146373000	-2.437118000
C	-4.353829000	1.969667000	-3.543661000
H	-3.731241000	2.564028000	-2.858244000
H	-5.127984000	2.632917000	-3.963710000
H	-3.716853000	1.623342000	-4.373386000
C	-5.806102000	-0.107040000	-3.783196000
H	-6.615092000	0.461557000	-4.269701000
H	-6.258416000	-0.962965000	-3.259186000
H	-5.154914000	-0.500530000	-4.579868000
C	2.664234000	-2.683025000	-0.449206000
C	2.292726000	-3.531036000	0.610541000
C	1.958321000	-4.849947000	0.279554000
H	1.650270000	-5.542202000	1.063363000
C	2.000320000	-5.290960000	-1.044262000
H	1.730299000	-6.323708000	-1.279743000
C	2.371732000	-4.422082000	-2.069044000
H	2.383995000	-4.779408000	-3.100279000
C	2.708292000	-3.088450000	-1.794942000
C	2.278444000	-3.024664000	2.046657000
H	1.921547000	-1.983316000	2.009362000
C	1.308375000	-3.790680000	2.951666000
H	1.231805000	-3.287464000	3.928140000
H	1.651090000	-4.820894000	3.144150000
H	0.302488000	-3.837613000	2.510332000
C	3.701637000	-3.021734000	2.637665000
H	4.399155000	-2.443256000	2.014968000
H	4.091648000	-4.050305000	2.713808000
H	3.699620000	-2.582651000	3.648549000
C	3.016917000	-2.107175000	-2.920330000
H	3.540064000	-1.245050000	-2.483638000
C	1.709670000	-1.582020000	-3.541934000
H	1.924688000	-0.803322000	-4.291758000
H	1.050648000	-1.151639000	-2.772427000
H	1.161535000	-2.396538000	-4.041467000

C	3.951302000	-2.692563000	-3.987794000
H	4.216528000	-1.916434000	-4.723054000
H	3.475668000	-3.515798000	-4.544701000
H	4.883032000	-3.074845000	-3.542805000
C	5.402397000	1.059283000	0.305092000
C	5.824866000	1.237621000	1.633876000
C	7.040860000	1.908716000	1.827883000
H	7.401649000	2.082111000	2.843872000
C	7.793280000	2.358348000	0.742944000
H	8.738431000	2.879004000	0.916822000
C	7.349281000	2.147754000	-0.564565000
H	7.953011000	2.502625000	-1.401721000
C	6.136313000	1.494157000	-0.813622000
C	4.990806000	0.771356000	2.820630000
H	4.170712000	0.147076000	2.437543000
C	4.352191000	1.970719000	3.543653000
H	5.126130000	2.634080000	3.963928000
H	3.714807000	1.624587000	4.373146000
H	3.729926000	2.564898000	2.857787000
C	5.804494000	-0.105804000	3.784443000
H	6.257134000	-0.961844000	3.260902000
H	5.152968000	-0.499120000	4.580925000
H	6.613203000	0.463003000	4.271171000
C	5.630979000	1.251280000	-2.229095000
H	4.548144000	1.062658000	-2.165864000
C	5.812585000	2.469811000	-3.144306000
H	6.875616000	2.686052000	-3.338774000
H	5.352556000	3.368160000	-2.704208000
H	5.334367000	2.283903000	-4.118935000
C	6.285321000	-0.011454000	-2.817421000
H	7.377131000	0.117545000	-2.899783000
H	5.888774000	-0.222397000	-3.823240000
H	6.091986000	-0.887226000	-2.180037000
C	-4.134226000	5.772611000	-0.218908000
H	-4.021899000	6.522287000	0.582726000
H	-4.099462000	6.322496000	-1.176120000
H	-5.140275000	5.334106000	-0.128280000
C	4.134248000	5.772634000	0.216614000
H	4.018333000	6.525561000	-0.581414000
H	4.103537000	6.318541000	1.176268000
H	5.140001000	5.334708000	0.119979000

3(T1): E= -3077.816205

Au	-0.001241000	-0.355626000	-0.245480000
N	-0.024653000	1.859303000	-0.033874000
N	-2.920061000	-1.351343000	-0.005831000
N	-4.229051000	-0.986347000	0.050913000
N	-4.175954000	0.358341000	-0.144182000

N	2.932286000	-1.307455000	-0.079771000
N	4.204088000	-0.953423000	0.118780000
N	4.148514000	0.381126000	0.176055000
C	-1.120537000	2.610756000	-0.402749000
C	-2.477496000	2.205344000	-0.534132000
C	-3.391077000	3.222665000	-0.890331000
H	-4.438796000	2.968348000	-1.032813000
C	-3.019444000	4.567271000	-1.081141000
C	-1.676604000	4.940931000	-0.946253000
H	-1.373353000	5.981480000	-1.090301000
C	-0.726159000	3.959577000	-0.637610000
C	0.713530000	3.983852000	-0.481611000
C	1.671560000	4.994063000	-0.574909000
H	1.369192000	6.025413000	-0.778261000
C	3.029125000	4.673053000	-0.417278000
C	3.401228000	3.337430000	-0.195031000
H	4.463459000	3.122275000	-0.125718000
C	2.469177000	2.277647000	-0.084827000
C	1.098487000	2.637198000	-0.182566000
C	-2.866660000	0.818839000	-0.327848000
C	-2.055412000	-0.311765000	-0.247720000
C	2.860209000	0.874077000	0.006569000
C	2.053362000	-0.263387000	-0.154294000
C	-2.569348000	-2.699130000	0.319547000
C	-2.723658000	-3.129706000	1.657889000
C	-2.371360000	-4.453907000	1.951882000
H	-2.475509000	-4.827479000	2.971079000
C	-1.866711000	-5.300051000	0.963866000
H	-1.582992000	-6.324531000	1.219202000
C	-1.721499000	-4.847871000	-0.347293000
H	-1.327688000	-5.527460000	-1.102758000
C	-2.085986000	-3.543033000	-0.705520000
C	-3.208144000	-2.168864000	2.740766000
H	-3.984072000	-1.542113000	2.277343000
C	-3.855079000	-2.865522000	3.943373000
H	-4.307735000	-2.113738000	4.608708000
H	-3.119430000	-3.427206000	4.542559000
H	-4.646830000	-3.564674000	3.631782000
C	-2.071100000	-1.237483000	3.203719000
H	-1.616267000	-0.695641000	2.362542000
H	-1.276414000	-1.811749000	3.704430000
H	-2.453256000	-0.491081000	3.918277000
C	-2.040266000	-3.062364000	-2.153027000
H	-1.642769000	-2.035661000	-2.144795000
C	-3.462520000	-3.000426000	-2.744351000
H	-4.126360000	-2.363572000	-2.143238000
H	-3.908345000	-4.008137000	-2.785861000
H	-3.435231000	-2.597132000	-3.770105000
C	-1.113567000	-3.890031000	-3.048702000

H	-1.023218000	-3.411618000	-4.036404000
H	-1.503849000	-4.907745000	-3.215287000
H	-0.105840000	-3.974877000	-2.617333000
C	-5.390608000	1.081405000	0.065618000
C	-5.597449000	1.724617000	1.301937000
C	-6.784069000	2.455656000	1.459784000
H	-6.969992000	2.979879000	2.400258000
C	-7.731889000	2.516178000	0.438888000
H	-8.651900000	3.088590000	0.583834000
C	-7.514107000	1.845293000	-0.766367000
H	-8.268725000	1.895447000	-1.553858000
C	-6.335861000	1.118511000	-0.980671000
C	-4.587036000	1.642264000	2.439201000
H	-3.753003000	1.008448000	2.108356000
C	-4.000762000	3.022266000	2.781126000
H	-3.225027000	2.927650000	3.558147000
H	-4.778199000	3.703732000	3.164815000
H	-3.543938000	3.488588000	1.895830000
C	-5.203138000	0.966720000	3.676008000
H	-4.448544000	0.858978000	4.472003000
H	-5.588915000	-0.035166000	3.431803000
H	-6.036925000	1.558835000	4.086879000
C	-6.085257000	0.378241000	-2.287385000
H	-5.005248000	0.173689000	-2.343326000
C	-6.454089000	1.206857000	-3.525822000
H	-5.950938000	2.186792000	-3.516746000
H	-7.539765000	1.382443000	-3.598340000
H	-6.149487000	0.675538000	-4.441703000
C	-6.807140000	-0.980911000	-2.276687000
H	-7.899822000	-0.842087000	-2.217839000
H	-6.486705000	-1.581140000	-1.412789000
H	-6.582770000	-1.547638000	-3.195150000
C	2.614879000	-2.695302000	-0.279733000
C	2.137243000	-3.440636000	0.817013000
C	1.792825000	-4.776209000	0.574292000
H	1.398057000	-5.390017000	1.383570000
C	1.933975000	-5.333276000	-0.697820000
H	1.651948000	-6.375961000	-0.864926000
C	2.420745000	-4.570463000	-1.758474000
H	2.515899000	-5.021951000	-2.747691000
C	2.770563000	-3.225126000	-1.573867000
C	2.047904000	-2.814528000	2.204461000
H	1.706527000	-1.774543000	2.067646000
C	1.032889000	-3.502783000	3.121921000
H	0.916717000	-2.924271000	4.051553000
H	1.364074000	-4.513829000	3.410582000
H	0.047630000	-3.588967000	2.643811000
C	3.438323000	-2.770472000	2.869421000
H	4.174385000	-2.247041000	2.243234000

H	3.810166000	-3.793159000	3.046522000
H	3.383961000	-2.254689000	3.841812000
C	3.232932000	-2.356929000	-2.738445000
H	3.741028000	-1.477931000	-2.317273000
C	2.023687000	-1.849144000	-3.544402000
H	2.348631000	-1.146169000	-4.328591000
H	1.301933000	-1.330306000	-2.895189000
H	1.498225000	-2.685637000	-4.031414000
C	4.252817000	-3.063206000	-3.641775000
H	4.632153000	-2.362852000	-4.402829000
H	3.806993000	-3.915245000	-4.179708000
H	5.111579000	-3.435961000	-3.062491000
C	5.378603000	1.054463000	0.483608000
C	5.643119000	1.378050000	1.826440000
C	6.840718000	2.057465000	2.094387000
H	7.078352000	2.342507000	3.121444000
C	7.730430000	2.373879000	1.068358000
H	8.658769000	2.902711000	1.299072000
C	7.445578000	2.019726000	-0.252604000
H	8.155987000	2.272259000	-1.041891000
C	6.257351000	1.353056000	-0.576030000
C	4.664404000	1.047416000	2.946058000
H	3.890803000	0.382674000	2.535295000
C	3.958144000	2.319400000	3.447919000
H	4.682403000	3.022110000	3.892285000
H	3.212799000	2.067628000	4.219380000
H	3.441554000	2.838279000	2.626772000
C	5.340092000	0.282396000	4.094560000
H	5.836351000	-0.629583000	3.728946000
H	4.589590000	-0.014461000	4.844444000
H	6.094344000	0.898602000	4.610100000
C	5.924712000	0.956151000	-2.008157000
H	4.837078000	0.792189000	-2.059908000
C	6.255580000	2.056582000	-3.025455000
H	7.340740000	2.227326000	-3.112103000
H	5.778647000	3.010157000	-2.749906000
H	5.891165000	1.769357000	-4.024452000
C	6.605471000	-0.377207000	-2.365724000
H	7.702791000	-0.279226000	-2.322676000
H	6.330129000	-0.693159000	-3.384701000
H	6.302876000	-1.169580000	-1.665386000
C	-4.085187000	5.576121000	-1.439938000
H	-3.676003000	6.595156000	-1.510065000
H	-4.554047000	5.334954000	-2.409479000
H	-4.893774000	5.585134000	-0.689752000
C	4.084287000	5.752479000	-0.482198000
H	3.878489000	6.469925000	-1.292983000
H	4.121832000	6.331095000	0.457785000
H	5.087880000	5.330859000	-0.646959000

4: E= -3117.562644

Au	0.003635000	0.541783000	-0.569090000
N	0.078588000	-2.093600000	0.245848000
N	-2.930732000	1.274168000	-0.099288000
N	-4.120169000	0.865317000	0.289105000
N	-4.012080000	-0.444141000	0.348675000
N	2.926784000	1.366427000	-0.015598000
N	4.152583000	0.949952000	0.238908000
N	4.092527000	-0.354990000	0.098242000
C	-1.064957000	-2.765191000	-0.213932000
C	-2.401842000	-2.312585000	-0.233864000
C	-3.349416000	-3.227474000	-0.741515000
H	-4.395726000	-2.935204000	-0.785228000
C	-3.004916000	-4.483576000	-1.272098000
C	-1.649193000	-4.824741000	-1.384883000
H	-1.354559000	-5.748464000	-1.889387000
C	-0.681041000	-3.957547000	-0.876334000
C	0.775174000	-3.917614000	-0.979551000
C	1.702533000	-4.723691000	-1.642617000
H	1.384407000	-5.655201000	-2.117850000
C	3.039076000	-4.307867000	-1.731760000
C	3.394362000	-3.047765000	-1.219200000
H	4.406216000	-2.694223000	-1.405395000
C	2.489056000	-2.200938000	-0.547068000
C	1.186954000	-2.712077000	-0.358397000
C	-2.755715000	-0.893221000	-0.014897000
C	-2.017168000	0.274183000	-0.301288000
C	2.832069000	-0.793635000	-0.259577000
C	2.043172000	0.369166000	-0.345510000
C	-2.696674000	2.674230000	-0.369068000
C	-2.782708000	3.095548000	-1.707376000
C	-2.475162000	4.438672000	-1.970670000
H	-2.523917000	4.808734000	-2.996737000
C	-2.105956000	5.302351000	-0.941725000
H	-1.865603000	6.344134000	-1.167484000
C	-2.040020000	4.848737000	0.377319000
H	-1.746111000	5.543148000	1.164313000
C	-2.333753000	3.517854000	0.699130000
C	-3.166720000	2.155281000	-2.843277000
H	-3.439671000	1.182062000	-2.409152000
C	-4.401151000	2.662250000	-3.606911000
H	-4.701812000	1.931393000	-4.374556000
H	-4.199516000	3.615715000	-4.120192000
H	-5.253537000	2.817459000	-2.928030000
C	-1.973583000	1.911417000	-3.782820000
H	-1.105756000	1.521614000	-3.227994000
H	-1.663416000	2.842602000	-4.283199000

H	-2.240122000	1.182102000	-4.564137000
C	-2.287942000	3.006429000	2.134416000
H	-1.940501000	1.959065000	2.092519000
C	-3.692637000	3.016846000	2.770543000
H	-4.412167000	2.427881000	2.185743000
H	-4.073695000	4.048616000	2.837035000
H	-3.654146000	2.602804000	3.791057000
C	-1.299666000	3.778356000	3.017104000
H	-1.191269000	3.274211000	3.989855000
H	-1.655889000	4.799661000	3.225779000
H	-0.308081000	3.852680000	2.549177000
C	-5.149009000	-1.184801000	0.842110000
C	-6.236805000	-1.390562000	-0.029548000
C	-7.323953000	-2.111126000	0.479718000
H	-8.193028000	-2.295495000	-0.153721000
C	-7.311222000	-2.600836000	1.787658000
H	-8.168436000	-3.165502000	2.161973000
C	-6.217797000	-2.370490000	2.621580000
H	-6.229851000	-2.753788000	3.643924000
C	-5.106321000	-1.644122000	2.169982000
C	-6.240329000	-0.840650000	-1.451920000
H	-5.194043000	-0.642583000	-1.737170000
C	-6.799204000	-1.837792000	-2.477375000
H	-6.668987000	-1.442460000	-3.496591000
H	-7.876335000	-2.015594000	-2.333997000
H	-6.285043000	-2.810074000	-2.422091000
C	-6.992835000	0.501620000	-1.510847000
H	-6.964651000	0.913233000	-2.532146000
H	-6.545580000	1.238140000	-0.827840000
H	-8.048875000	0.366941000	-1.226543000
C	-3.925624000	-1.373900000	3.095836000
H	-3.227888000	-0.701154000	2.574413000
C	-3.161479000	-2.670791000	3.413445000
H	-2.817266000	-3.167926000	2.493897000
H	-3.799220000	-3.381372000	3.963151000
H	-2.281456000	-2.456463000	4.041186000
C	-4.364599000	-0.642240000	4.374879000
H	-5.032047000	-1.263962000	4.991880000
H	-4.895091000	0.292311000	4.136603000
H	-3.486138000	-0.390234000	4.990181000
C	2.592841000	2.756477000	0.210258000
C	2.241238000	3.553448000	-0.896809000
C	1.862873000	4.875944000	-0.627431000
H	1.575057000	5.532649000	-1.448039000
C	1.851889000	5.369772000	0.676195000
H	1.556582000	6.405531000	0.860590000
C	2.215825000	4.554197000	1.747388000
H	2.200506000	4.961415000	2.758584000
C	2.588209000	3.218657000	1.544773000

C	2.323532000	3.020896000	-2.323124000
H	1.983859000	1.975139000	-2.303470000
C	1.405243000	3.756617000	-3.305601000
H	1.391793000	3.227858000	-4.271042000
H	1.750410000	4.783908000	-3.504304000
H	0.371926000	3.805149000	-2.929492000
C	3.785511000	3.023464000	-2.810003000
H	4.438168000	2.440886000	-2.142579000
H	4.181620000	4.051013000	-2.849442000
H	3.857198000	2.591084000	-3.820926000
C	2.901413000	2.295027000	2.721170000
H	3.668346000	1.583809000	2.385238000
C	1.652819000	1.482962000	3.116900000
H	1.921363000	0.675661000	3.817020000
H	1.163106000	1.037336000	2.239543000
H	0.912175000	2.127134000	3.612190000
C	3.490698000	3.016694000	3.938887000
H	3.810118000	2.277206000	4.689525000
H	2.754476000	3.674485000	4.427651000
H	4.365708000	3.625683000	3.665084000
C	5.265075000	-1.126582000	0.438750000
C	6.303055000	-1.216932000	-0.508464000
C	7.422621000	-1.976218000	-0.146436000
H	8.256797000	-2.073691000	-0.843003000
C	7.486241000	-2.614349000	1.094170000
H	8.366889000	-3.206965000	1.353130000
C	6.438920000	-2.496951000	2.007176000
H	6.509539000	-2.996257000	2.975740000
C	5.299226000	-1.738242000	1.704336000
C	6.224672000	-0.496442000	-1.849114000
H	5.162449000	-0.283351000	-2.053917000
C	6.742158000	-1.347460000	-3.017708000
H	7.827415000	-1.521364000	-2.951269000
H	6.554202000	-0.831954000	-3.972048000
H	6.244195000	-2.329281000	-3.057351000
C	6.956091000	0.856577000	-1.771554000
H	6.545243000	1.483512000	-0.966752000
H	6.858050000	1.403534000	-2.722528000
H	8.029433000	0.706087000	-1.572790000
C	4.171244000	-1.594267000	2.719493000
H	3.421446000	-0.904168000	2.305981000
C	3.465690000	-2.938124000	2.968172000
H	4.153487000	-3.671285000	3.418594000
H	3.080234000	-3.366413000	2.030580000
H	2.619138000	-2.805629000	3.660952000
C	4.669741000	-0.960147000	4.028627000
H	5.400430000	-1.605115000	4.541272000
H	3.827312000	-0.800233000	4.720398000
H	5.150514000	0.012780000	3.842691000

C	-4.084501000	-5.422466000	-1.752871000
H	-3.724182000	-6.076369000	-2.561043000
H	-4.429636000	-6.074760000	-0.932212000
H	-4.964890000	-4.875437000	-2.123523000
C	4.079327000	-5.171660000	-2.402560000
H	4.563497000	-5.841245000	-1.670845000
H	3.636893000	-5.806660000	-3.184644000
H	4.874478000	-4.565810000	-2.863734000
C	0.144460000	-1.694073000	1.654028000
H	-0.698227000	-1.036472000	1.902156000
H	0.115987000	-2.580983000	2.310477000
H	1.071332000	-1.143139000	1.841090000

4(T1): E= -3117.476191

Au	0.031421000	0.503051000	-0.409901000
N	0.082027000	-2.115964000	0.459684000
N	-2.884926000	1.285736000	-0.239328000
N	-4.148781000	0.962010000	0.032551000
N	-4.103598000	-0.346211000	0.279140000
N	2.992561000	1.302657000	0.037581000
N	4.216846000	0.860661000	0.258250000
N	4.122286000	-0.444389000	0.131231000
C	-1.096624000	-2.731951000	0.063436000
C	-2.465921000	-2.248216000	0.228106000
C	-3.464604000	-3.322457000	0.081747000
H	-4.474719000	-3.167926000	0.450624000
C	-3.180580000	-4.476292000	-0.607577000
C	-1.846170000	-4.734007000	-1.107714000
H	-1.639292000	-5.642656000	-1.675558000
C	-0.809344000	-3.861417000	-0.715251000
C	0.627850000	-3.854515000	-0.928509000
C	1.487506000	-4.651182000	-1.695196000
H	1.105584000	-5.536013000	-2.210007000
C	2.832782000	-4.288328000	-1.822145000
C	3.275491000	-3.075853000	-1.249994000
H	4.288013000	-2.747020000	-1.476087000
C	2.446281000	-2.242584000	-0.480012000
C	1.135899000	-2.716203000	-0.245527000
C	-2.796794000	-0.872625000	0.165101000
C	-1.995353000	0.271051000	-0.185310000
C	2.842694000	-0.853075000	-0.180142000
C	2.077654000	0.323502000	-0.250203000
C	-2.576018000	2.643165000	-0.624335000
C	-2.621671000	2.961639000	-1.991846000
C	-2.262027000	4.269249000	-2.351306000
H	-2.276986000	4.560349000	-3.403402000
C	-1.877903000	5.196310000	-1.384532000
H	-1.596353000	6.208495000	-1.685121000

C	-1.846530000	4.843186000	-0.033698000
H	-1.537909000	5.584813000	0.703137000
C	-2.198571000	3.553328000	0.383019000
C	-2.993907000	1.937434000	-3.056712000
H	-3.370636000	1.036435000	-2.550392000
C	-4.124589000	2.436670000	-3.969622000
H	-4.420078000	1.644125000	-4.675156000
H	-3.813902000	3.308608000	-4.566761000
H	-5.012309000	2.722875000	-3.385716000
C	-1.751487000	1.524610000	-3.865414000
H	-0.961080000	1.132430000	-3.206150000
H	-1.334959000	2.383153000	-4.416211000
H	-2.008450000	0.743898000	-4.598973000
C	-2.207296000	3.152073000	1.853501000
H	-1.893523000	2.095001000	1.901900000
C	-3.630276000	3.249033000	2.439054000
H	-4.348167000	2.644041000	1.867659000
H	-3.979792000	4.293952000	2.423940000
H	-3.640811000	2.901487000	3.484372000
C	-1.216996000	3.954262000	2.705031000
H	-1.163754000	3.528952000	3.719209000
H	-1.532543000	5.004017000	2.815186000
H	-0.207506000	3.945903000	2.270073000
C	-5.321265000	-0.961081000	0.730861000
C	-6.246842000	-1.397018000	-0.238629000
C	-7.422661000	-1.995904000	0.229162000
H	-8.169982000	-2.349042000	-0.483017000
C	-7.649374000	-2.155601000	1.598589000
H	-8.570514000	-2.632465000	1.942290000
C	-6.712480000	-1.710190000	2.530879000
H	-6.909288000	-1.842315000	3.596619000
C	-5.525102000	-1.089001000	2.117100000
C	-5.970341000	-1.214546000	-1.725164000
H	-4.877610000	-1.133693000	-1.845269000
C	-6.422308000	-2.411929000	-2.571970000
H	-6.092124000	-2.282338000	-3.614422000
H	-7.518913000	-2.513659000	-2.590868000
H	-5.997447000	-3.354573000	-2.193712000
C	-6.589627000	0.101647000	-2.230698000
H	-6.344247000	0.262119000	-3.292577000
H	-6.215097000	0.961121000	-1.655766000
H	-7.687103000	0.077075000	-2.133705000
C	-4.489976000	-0.613918000	3.130255000
H	-3.770890000	0.028828000	2.599802000
C	-3.706320000	-1.805952000	3.708707000
H	-3.225037000	-2.395874000	2.913333000
H	-4.374577000	-2.481419000	4.266905000
H	-2.924151000	-1.456763000	4.402546000
C	-5.110363000	0.244807000	4.242581000

H	-5.795238000	-0.338119000	4.878104000
H	-5.672436000	1.093609000	3.824995000
H	-4.320432000	0.646641000	4.896835000
C	2.698084000	2.705073000	0.231342000
C	2.363202000	3.482444000	-0.894992000
C	2.030554000	4.822892000	-0.656971000
H	1.757227000	5.467521000	-1.492022000
C	2.047114000	5.350151000	0.634093000
H	1.788626000	6.399846000	0.793192000
C	2.386415000	4.549897000	1.724594000
H	2.385302000	4.981205000	2.726191000
C	2.711885000	3.197633000	1.553318000
C	2.412936000	2.906237000	-2.306230000
H	2.039080000	1.872960000	-2.252625000
C	1.508651000	3.642169000	-3.301186000
H	1.474978000	3.089694000	-4.252755000
H	1.880979000	4.654111000	-3.527671000
H	0.479717000	3.729074000	-2.920853000
C	3.868663000	2.847191000	-2.808196000
H	4.511304000	2.265105000	-2.131014000
H	4.295821000	3.860140000	-2.883201000
H	3.914618000	2.382847000	-3.806344000
C	2.981875000	2.289879000	2.751393000
H	3.652684000	1.486956000	2.417601000
C	1.672286000	1.634145000	3.231994000
H	1.883421000	0.842397000	3.969217000
H	1.107757000	1.193403000	2.396679000
H	1.018381000	2.377775000	3.711422000
C	3.700377000	2.993955000	3.909256000
H	3.974547000	2.257659000	4.680675000
H	3.061110000	3.747818000	4.395091000
H	4.620606000	3.492695000	3.569023000
C	5.279413000	-1.248895000	0.446663000
C	6.290850000	-1.377372000	-0.524222000
C	7.395592000	-2.166997000	-0.181620000
H	8.209883000	-2.294817000	-0.896623000
C	7.470803000	-2.794915000	1.063471000
H	8.340052000	-3.410410000	1.307050000
C	6.450495000	-2.637713000	2.000956000
H	6.531179000	-3.128187000	2.973281000
C	5.326402000	-1.849143000	1.717559000
C	6.206358000	-0.659598000	-1.865915000
H	5.148350000	-0.406378000	-2.045788000
C	6.664413000	-1.532497000	-3.043249000
H	7.743825000	-1.745677000	-3.001216000
H	6.473421000	-1.012963000	-3.994811000
H	6.131324000	-2.496209000	-3.068295000
C	6.990237000	0.664991000	-1.810947000
H	6.623805000	1.309394000	-0.998488000

H	6.889572000	1.212761000	-2.761090000
H	8.061637000	0.474344000	-1.637979000
C	4.231330000	-1.656290000	2.760337000
H	3.481822000	-0.964606000	2.348797000
C	3.508171000	-2.978306000	3.067116000
H	4.195045000	-3.714977000	3.512893000
H	3.082758000	-3.422339000	2.153974000
H	2.688972000	-2.811506000	3.784931000
C	4.780509000	-0.993627000	4.034717000
H	5.510819000	-1.639816000	4.546471000
H	3.961836000	-0.791172000	4.743912000
H	5.277988000	-0.038761000	3.804358000
C	-4.245844000	-5.507103000	-0.865188000
H	-4.397668000	-5.640392000	-1.950047000
H	-3.945897000	-6.490842000	-0.465592000
H	-5.208543000	-5.227909000	-0.414331000
C	3.805711000	-5.140229000	-2.598242000
H	4.460660000	-5.705498000	-1.913203000
H	3.289151000	-5.868197000	-3.240334000
H	4.460803000	-4.525479000	-3.235990000
C	0.234165000	-1.649345000	1.841694000
H	-0.639211000	-1.048297000	2.123189000
H	0.309561000	-2.518804000	2.515200000
H	1.134904000	-1.037561000	1.943827000

5: E= -3177.470576

Au	0.000047000	-0.219890000	-0.001436000
N	-0.000128000	1.790458000	-0.001209000
N	2.906756000	-1.245722000	-0.359918000
N	4.173727000	-0.909845000	-0.274916000
N	4.171182000	0.376738000	0.006723000
N	-2.906382000	-1.245955000	0.359139000
N	-4.173466000	-0.910009000	0.275715000
N	-4.171165000	0.376520000	-0.006154000
C	1.116449000	2.607769000	0.132889000
C	2.489707000	2.278319000	0.255626000
C	3.393745000	3.346299000	0.426464000
H	4.451911000	3.129732000	0.547199000
C	3.001091000	4.694290000	0.434091000
C	1.647347000	5.000741000	0.246615000
H	1.318727000	6.043057000	0.219518000
C	0.715113000	3.972486000	0.099022000
C	-0.715584000	3.972328000	-0.103407000
C	-1.647925000	5.000361000	-0.251970000
H	-1.319412000	6.042733000	-0.225767000
C	-3.001601000	4.693602000	-0.439370000
C	-3.394138000	3.345563000	-0.430537000
H	-4.452269000	3.128803000	-0.551256000

C	-2.490011000	2.277860000	-0.258457000
C	-1.116780000	2.607557000	-0.136059000
C	2.893330000	0.889507000	0.098001000
C	2.053543000	-0.202670000	-0.124651000
C	-2.893426000	0.889141000	-0.099453000
C	-2.053415000	-0.203005000	0.122491000
C	2.610175000	-2.606344000	-0.759322000
C	2.475310000	-2.865974000	-2.132122000
C	2.211355000	-4.193950000	-2.495056000
H	2.088742000	-4.449848000	-3.548905000
C	2.096668000	-5.191416000	-1.527009000
H	1.894579000	-6.220797000	-1.832756000
C	2.226867000	-4.885694000	-0.171468000
H	2.119576000	-5.677855000	0.571219000
C	2.478512000	-3.574106000	0.247153000
C	2.540940000	-1.762703000	-3.181910000
H	2.976516000	-0.866581000	-2.713771000
C	3.446749000	-2.125054000	-4.368066000
H	3.530538000	-1.269935000	-5.057125000
H	3.043159000	-2.970973000	-4.945918000
H	4.459076000	-2.394931000	-4.031854000
C	1.123842000	-1.389427000	-3.654291000
H	0.473614000	-1.126152000	-2.806222000
H	0.651832000	-2.235578000	-4.177195000
H	1.156886000	-0.533593000	-4.347169000
C	2.598449000	-3.211868000	1.719890000
H	2.459166000	-2.122777000	1.807994000
C	4.005681000	-3.539013000	2.249024000
H	4.781130000	-3.020988000	1.663604000
H	4.204614000	-4.621142000	2.188564000
H	4.107103000	-3.233163000	3.302808000
C	1.492669000	-3.858320000	2.563960000
H	1.534325000	-3.479960000	3.597641000
H	1.600814000	-4.953276000	2.614866000
H	0.504643000	-3.626704000	2.142727000
C	5.449910000	1.015881000	0.207727000
C	6.064426000	1.624288000	-0.903358000
C	7.293473000	2.253813000	-0.672369000
H	7.815430000	2.740745000	-1.497144000
C	7.861992000	2.269013000	0.604045000
H	8.819907000	2.770363000	0.761911000
C	7.221704000	1.650484000	1.677529000
H	7.682581000	1.674924000	2.666664000
C	5.992093000	0.998348000	1.504248000
C	5.426663000	1.572827000	-2.285721000
H	4.338270000	1.466371000	-2.145710000
C	5.640370000	2.857087000	-3.097135000
H	5.057552000	2.813868000	-4.030166000
H	6.695474000	2.993469000	-3.381975000

H	5.318243000	3.746783000	-2.533871000
C	5.917352000	0.330190000	-3.052709000
H	5.421487000	0.259999000	-4.033367000
H	5.708374000	-0.594597000	-2.494463000
H	7.004615000	0.384226000	-3.222337000
C	5.269304000	0.356657000	2.682379000
H	4.440730000	-0.251000000	2.287971000
C	4.654789000	1.437267000	3.590705000
H	3.962479000	2.087562000	3.033461000
H	5.439845000	2.076207000	4.026317000
H	4.097470000	0.973568000	4.420144000
C	6.177566000	-0.598902000	3.471222000
H	7.001694000	-0.063851000	3.968660000
H	6.616003000	-1.366857000	2.815958000
H	5.597835000	-1.110132000	4.255505000
C	-2.609405000	-2.606553000	0.758441000
C	-2.478476000	-3.574479000	-0.247949000
C	-2.226841000	-4.886092000	0.170660000
H	-2.120231000	-5.678312000	-0.572083000
C	-2.095837000	-5.191712000	1.526114000
H	-1.893795000	-6.221100000	1.831866000
C	-2.209641000	-4.194065000	2.494102000
H	-2.086350000	-4.449860000	3.547893000
C	-2.473580000	-2.866089000	2.131203000
C	-2.598730000	-3.212804000	-1.720779000
H	-2.463195000	-2.123275000	-1.809077000
C	-1.489928000	-3.855680000	-2.563693000
H	-1.532068000	-3.477954000	-3.597592000
H	-1.594087000	-4.951050000	-2.614093000
H	-0.503156000	-3.620351000	-2.141620000
C	-4.004367000	-3.544989000	-2.250879000
H	-4.782015000	-3.029431000	-1.666208000
H	-4.199657000	-4.627775000	-2.190294000
H	-4.106047000	-3.239781000	-3.304819000
C	-2.538094000	-1.762718000	3.180967000
H	-2.974412000	-0.866716000	2.713290000
C	-1.120450000	-1.389184000	3.651532000
H	-1.152727000	-0.533152000	4.344201000
H	-0.471258000	-1.126107000	2.802616000
H	-0.647764000	-2.235149000	4.174121000
C	-3.442245000	-2.125130000	4.368361000
H	-3.525253000	-1.269978000	5.057470000
H	-3.037687000	-2.970907000	4.945739000
H	-4.454972000	-2.395235000	4.033554000
C	-5.450037000	1.016273000	-0.204135000
C	-5.994984000	0.999945000	-1.499436000
C	-7.224763000	1.652599000	-1.669710000
H	-7.687681000	1.677652000	-2.657904000
C	-7.862453000	2.270833000	-0.594546000

H	-8.820503000	2.772672000	-0.750012000
C	-7.291045000	2.254750000	0.680595000
H	-7.810902000	2.741512000	1.506800000
C	-6.061819000	1.624526000	0.908574000
C	-5.275975000	0.357992000	-2.679665000
H	-4.440178000	-0.241883000	-2.288562000
C	-4.676007000	1.438312000	-3.597888000
H	-5.468769000	2.068709000	-4.031963000
H	-4.120466000	0.974475000	-4.428431000
H	-3.985185000	2.096938000	-3.048589000
C	-6.183962000	-0.607672000	-3.456614000
H	-6.611100000	-1.375901000	-2.794255000
H	-5.607477000	-1.118147000	-4.243792000
H	-7.016412000	-0.080611000	-3.948725000
C	-5.421340000	1.571923000	2.289620000
H	-4.333398000	1.463701000	2.147513000
C	-5.631371000	2.856474000	3.101559000
H	-6.685717000	2.994680000	3.388319000
H	-5.308732000	3.745650000	2.537767000
H	-5.046940000	2.812163000	4.033533000
C	-5.912573000	0.329950000	3.057311000
H	-6.999447000	0.385612000	3.228889000
H	-5.415070000	0.258807000	4.037061000
H	-5.705977000	-0.595021000	2.498470000
C	4.026853000	5.780039000	0.653969000
H	3.729290000	6.721746000	0.168783000
H	4.153847000	5.992008000	1.729761000
H	5.013248000	5.490156000	0.260428000
C	-4.027483000	5.779008000	-0.660394000
H	-3.729644000	6.721484000	-0.176878000
H	-4.155113000	5.989258000	-1.736448000
H	-5.013645000	5.489741000	-0.265809000
F	0.000162000	-2.184125000	-0.001629000

5(T1): E= -3177.409862

Au	0.000018000	-0.395664000	-0.000003000
N	0.000021000	1.908534000	-0.000026000
N	2.999102000	-1.248827000	-0.253476000
N	4.250786000	-0.864214000	-0.110656000
N	4.191531000	0.429167000	0.087273000
N	-2.999099000	-1.248812000	0.253470000
N	-4.250772000	-0.864181000	0.110593000
N	-4.191491000	0.429190000	-0.087393000
C	1.110022000	2.700560000	0.072923000
C	2.479221000	2.310574000	0.148115000
C	3.405692000	3.373968000	0.241359000
H	4.465099000	3.151155000	0.309430000
C	3.040875000	4.727587000	0.242620000

C	1.673112000	5.085951000	0.139481000
H	1.382507000	6.139726000	0.128269000
C	0.729889000	4.083373000	0.055870000
C	-0.729850000	4.083371000	-0.055964000
C	-1.673077000	5.085946000	-0.139601000
H	-1.382476000	6.139721000	-0.128392000
C	-3.040836000	4.727576000	-0.242756000
C	-3.405650000	3.373954000	-0.241474000
H	-4.465057000	3.151133000	-0.309550000
C	-2.479177000	2.310569000	-0.148203000
C	-1.109979000	2.700558000	-0.072994000
C	2.885137000	0.901423000	0.069773000
C	2.086748000	-0.237829000	-0.130707000
C	-2.885095000	0.901422000	-0.069849000
C	-2.086720000	-0.237838000	0.130648000
C	2.759845000	-2.635922000	-0.597719000
C	2.803806000	-2.979119000	-1.959005000
C	2.564768000	-4.323399000	-2.274422000
H	2.577605000	-4.644486000	-3.317072000
C	2.305148000	-5.257027000	-1.271130000
H	2.125476000	-6.301184000	-1.538894000
C	2.259392000	-4.869586000	0.067636000
H	2.032911000	-5.611911000	0.833953000
C	2.471816000	-3.537028000	0.437256000
C	3.016875000	-1.936468000	-3.051427000
H	3.518634000	-1.065865000	-2.601542000
C	3.929303000	-2.427606000	-4.183650000
H	4.133720000	-1.607063000	-4.889637000
H	3.463179000	-3.241081000	-4.761042000
H	4.891532000	-2.794247000	-3.795005000
C	1.660573000	-1.453115000	-3.598285000
H	1.019278000	-1.061931000	-2.793883000
H	1.118545000	-2.283624000	-4.076345000
H	1.803340000	-0.657901000	-4.348110000
C	2.392626000	-3.082657000	1.886731000
H	2.166679000	-2.005041000	1.880633000
C	3.749386000	-3.268163000	2.589437000
H	4.552732000	-2.731785000	2.059857000
H	4.028088000	-4.333685000	2.627093000
H	3.707730000	-2.891585000	3.624578000
C	1.243631000	-3.755041000	2.648291000
H	1.140617000	-3.304561000	3.648510000
H	1.422286000	-4.831880000	2.797751000
H	0.304651000	-3.621557000	2.093067000
C	5.444660000	1.099188000	0.350431000
C	6.180117000	1.579885000	-0.749812000
C	7.378408000	2.244431000	-0.461581000
H	7.989303000	2.635504000	-1.276522000
C	7.805273000	2.412109000	0.858541000

H	8.741883000	2.937352000	1.060501000
C	7.053346000	1.908042000	1.919674000
H	7.408167000	2.041162000	2.943481000
C	5.849072000	1.226214000	1.690534000
C	5.702820000	1.356995000	-2.178892000
H	4.611425000	1.204282000	-2.145719000
C	5.953451000	2.563499000	-3.093451000
H	5.484316000	2.395501000	-4.075133000
H	7.027577000	2.727749000	-3.273033000
H	5.533016000	3.487902000	-2.666862000
C	6.329450000	0.071943000	-2.752380000
H	5.948293000	-0.124875000	-3.766337000
H	6.099009000	-0.800388000	-2.123023000
H	7.425406000	0.168981000	-2.810799000
C	5.021346000	0.687509000	2.851236000
H	4.205085000	0.074870000	2.439723000
C	4.374968000	1.838769000	3.642138000
H	3.741803000	2.465600000	2.994924000
H	5.142511000	2.487725000	4.093831000
H	3.747171000	1.442305000	4.455597000
C	5.846771000	-0.234910000	3.762091000
H	6.650940000	0.313143000	4.278207000
H	6.305537000	-1.054744000	3.189056000
H	5.200786000	-0.679504000	4.535046000
C	-2.759921000	-2.635895000	0.597825000
C	-2.472005000	-3.537100000	-0.437087000
C	-2.259713000	-4.869657000	-0.067380000
H	-2.033339000	-5.612060000	-0.833656000
C	-2.305478000	-5.257000000	1.271413000
H	-2.125917000	-6.301157000	1.539249000
C	-2.564989000	-4.323272000	2.274642000
H	-2.577847000	-4.644282000	3.317316000
C	-2.803903000	-2.978992000	1.959134000
C	-2.392755000	-3.082859000	-1.886596000
H	-2.167068000	-2.005186000	-1.880598000
C	-1.243505000	-3.755064000	-2.647938000
H	-1.140478000	-3.304682000	-3.648200000
H	-1.421895000	-4.831963000	-2.797285000
H	-0.304627000	-3.621312000	-2.092611000
C	-3.749390000	-3.268757000	-2.589436000
H	-4.552908000	-2.732487000	-2.060008000
H	-4.027853000	-4.334345000	-2.626989000
H	-3.707702000	-2.892300000	-3.624620000
C	-3.016908000	-1.936256000	3.051489000
H	-3.518499000	-1.065597000	2.601520000
C	-1.660580000	-1.453087000	3.598437000
H	-1.803286000	-0.657844000	4.348242000
H	-1.019185000	-1.062005000	2.794068000
H	-1.118692000	-2.283663000	4.076539000

C	-3.929510000	-2.427211000	4.183651000
H	-4.133884000	-1.606602000	4.889576000
H	-3.463553000	-3.240721000	4.761129000
H	-4.891751000	-2.793739000	3.794929000
C	-5.444601000	1.099259000	-0.350524000
C	-5.849134000	1.226184000	-1.690603000
C	-7.053372000	1.908095000	-1.919693000
H	-7.408292000	2.041124000	-2.943477000
C	-7.805135000	2.412357000	-0.858540000
H	-8.741716000	2.937669000	-1.060460000
C	-7.378148000	2.244791000	0.461558000
H	-7.988918000	2.636025000	1.276514000
C	-6.179899000	1.580150000	0.749741000
C	-5.021611000	0.687251000	-2.851346000
H	-4.205364000	0.074570000	-2.439873000
C	-4.375210000	1.838347000	-3.642471000
H	-5.142741000	2.487340000	-4.094132000
H	-3.747572000	1.441717000	-4.455972000
H	-3.741881000	2.465178000	-2.995418000
C	-5.847268000	-0.235171000	-3.761984000
H	-6.306057000	-1.054883000	-3.188794000
H	-5.201442000	-0.679931000	-4.534976000
H	-6.651441000	0.312922000	-4.278052000
C	-5.702466000	1.357351000	2.178791000
H	-4.611081000	1.204571000	2.145510000
C	-5.952921000	2.563939000	3.093287000
H	-7.027015000	2.728252000	3.272999000
H	-5.532498000	3.488289000	2.666571000
H	-5.483666000	2.395997000	4.074922000
C	-6.329113000	0.072383000	2.752455000
H	-7.425057000	0.169495000	2.810973000
H	-5.947867000	-0.124366000	3.766391000
H	-6.098792000	-0.800021000	2.123155000
C	4.089746000	5.801048000	0.349966000
H	4.023510000	6.502653000	-0.498195000
H	3.945152000	6.397517000	1.266730000
H	5.105646000	5.382158000	0.372312000
C	-4.089708000	5.801023000	-0.350242000
H	-4.022958000	6.503233000	0.497370000
H	-3.945601000	6.396822000	-1.267525000
H	-5.105643000	5.382172000	-0.371746000
F	0.000053000	-2.539043000	0.000167000

Section S7: References

1. D. I. Bezuidenhout, G. Kleinhans, G. Guisado-Barrios, D. C. Liles, G. Ung, G. Bertrand, *Chem. Commun.* **2014**, *50*, 2431-2433.
2. G. Kleinhans, M. M. Hansmann, G. Guisado-Barrios, D. C. Liles, G. Bertrand, D. I. Bezuidenhout, *J. Am. Chem. Soc.* **2016**, *138*, 15873-15876.
3. E. C. Keske, O. V. Zenkina, R. Wang, C. M. Crudden, *Organometallics*, **2012**, *31*, 456–461
4. BrukerAXS Inc., Madison, WI, 2015–2016.
5. G. M. Sheldrick, *ActaCrystallogr*., *Sect A: Fundam. Crystallogr*. **2015**, *71*, 3-8.
6. G. M. Sheldrick, *ActaCrystallogr*., *Sect C: Cryst. Struct. Commun.* **2015**, *71*, 3-8.
7. O.V. Dolomanov, L.J. Bourhis, R.J. Gildea, J.A.K Howard & H. Puschmann, *J. Appl. Cryst.* **2009**, *42*, 339-341.
8. A. L. Spek, *ActaCrystallogr*., *Sect C: Cryst. Struct. Commun*. **2015**, *71*, 9-18.
9. J. N. Demas, G. A. Crosby, *J. Phys. Chem*. **1971**, *75*, 991.
10. J. van Houten, R. Watts, *J. Am. Chem. Soc*. **1976**, *98*, 4853.
11. Gaussian 09, Revision D.01, M. J. Frisch, G. W. Trucks, H. B. Schlegel, G. E. Scuseria, M. A. Robb, J. R. Cheeseman, G. Scalmani, V. Barone, G. A. Petersson, H. Nakatsuji, X. Li, M. Caricato, A. Marenich, J. Bloino, B. G. Janesko, R. Gomperts, B. Mennucci, H. P. Hratchian, J. V. Ortiz, A. F. Izmaylov, J. L. Sonnenberg, D. Williams-Young, F. Ding, F. Lipparini, F. Egidi, J. Goings, B. Peng, A. Petrone, T. Henderson, D. Ranasinghe, V. G. Zakrzewski, J. Gao, N. Rega, G. Zheng, W. Liang, M. Hada, M. Ehara, K. Toyota, R. Fukuda, J. Hasegawa, M. Ishida, T. Nakajima, Y. Honda, O. Kitao, H. Nakai, T. Vreven, K. Throssell, J. A. Montgomery, Jr., J. E. Peralta, F. Ogliaro, M. Bearpark, J. J. Heyd, E. Brothers, K. N. Kudin, V. N. Staroverov, T. Keith, R. Kobayashi, J. Normand, K. Raghavachari, A. Rendell, J. C. Burant, S. S. Iyengar, J. Tomasi, M. Cossi, J. M. Millam, M. Klene, C. Adamo, R. Cammi, J. W. Ochterski, R. L. Martin, K. Morokuma, O. Farkas, J. B. Foresman, and D. J. Fox, Gaussian, Inc., Wallingford CT, **2016**.
12. a) A. D. Becke, *J. Chem. Phys*. **1993**, *98*, 5648; b) C. Lee, W. Yang, R. G. Parr, *Phys. Rev. B,* **1998**, *37*, 785; c) S. H. Vosko, L. Wilk, M. Nusair, *Can. J. Phys*. **1980**, *58*, 1200.
13. S. Grimme, J. Antony, S. Ehrlich, H. Krieg, H. *J. Chem. Phys*. **2010**, *132*, 154104.
14. F. Weigend, R. Alhrichs, *Phys. Chem. Chem. Phys*. **2005**, *7*, 3297.
15. J. W. McIver, A. K. Komornicki, *J. Am. Chem. Soc.* **1972**, *94*, 2625.
16. a) M. E. Casida, *Recent Developments and Applications of Modern Density Functional Theory*; Elsevier: Amsterdam, 1996; Vol. 4; b) M. E. Casida, D. P. Chong, *Recent Advances in Density Functional Methods*; World Scientific: Singapore, 1995; Vol. 1, p 155.
17. For a review, see: A. Dreuw, M. Head-Gordon, *Chem. Rev*. **2005**, *105*, 4009.
18. a) S. Miertuš, E. Scrocco, J. Tomasi, *Chem. Phys*. **1981**, *55*, 117; b) J. L. Pascual-Ahuir, E. Silla, I. Tuñón, *J. Comp. Chem*. **1994**, *15*, 1127; c) V. Barone, M. Cossi, *J. Phys. Chem. A*, **1998**, *102*, 1995.
